# The Super Enhancer‐Driven Long Noncoding RNA PRKCQ‐AS1 Promotes Neuroblastoma Tumorigenesis by Interacting With MSI2 Protein and Is Targetable by Small Molecule Compounds

**DOI:** 10.1002/advs.202412520

**Published:** 2025-03-18

**Authors:** Sujanna Mondal, Pei Y. Liu, Janith Seneviratne, Antoine De Weck, Pooja Venkat, Chelsea Mayoh, Jing Wu, Jesper Maag, Jingwei Chen, Matthew Wong, Nenad Bartonicek, Poh Khoo, Lei Jin, Louise E. Ludlow, David S. Ziegler, Toby Trahair, Pieter Mestdagh, Belamy B. Cheung, Jinyan Li, Marcel E. Dinger, Ian Street, Xu D. Zhang, Glenn M. Marshall, Tao Liu

**Affiliations:** ^1^ Children's Cancer Institute Australia and UNSW Centre for Childhood Cancer Research University of New South Wales Sydney NSW 2052 Australia; ^2^ Garvan Institute of Medical Research Genome Informatics Genomics & Epigenetics Division, 384 Victoria St. Darlinghurst NSW 2010 Australia; ^3^ School of Medicine and Public Health University of Newcastle Callaghan NSW 2308 Australia; ^4^ Translational Research Institute Henan Provincial People's Hospital Tianjian Laboratory of Advanced Biomedical Science Academy of Medical Sciences Zhengzhou University Zhengzhou China; ^5^ Murdoch Children's Research Institute The Royal Children's Hospital & Department of Paediatrics University of Melbourne Melbourne Australia; ^6^ Kids Cancer Centre Sydney Children's Hospital High Street Randwick NSW 2031 Australia; ^7^ Center for Medical Genetics Ghent Ghent University Ghent Belgium; ^8^ Shenzhen Institute of Advanced Technology Chinese Academy of Sciences Shenzhen China; ^9^ School of Life and Environmental Sciences Faculty of Science The University of Sydney Sydney NSW Australia; ^10^ School of Clinical Medicine UNSW Medicine & Health UNSW Sydney Kensington NSW Australia; ^11^ School of Medicine and Public Health Priority Research Centre for Cancer Research University of Newcastle Callaghan NSW 2308 Australia

**Keywords:** long noncoding RNA, long noncoding RNA inhibitor, Neuroblastoma, RNA binding protein, small molecule compounds, tumorigenesis

## Abstract

Tumorigenic drivers of *MYCN* gene nonamplified neuroblastoma remain largely uncharacterized. Long noncoding RNAs (lncRNAs) regulate tumorigenesis, however, there is little literature on therapeutic targeting of lncRNAs with small molecule compounds. Here PRKCQ‐AS1 is identified as the lncRNA most overexpressed in *MYCN* nonamplified, compared with *MYCN*‐amplified, neuroblastoma cell lines. *PRKCQ‐AS1* expression is controlled by super‐enhancers, and PRKCQ‐AS1 RNA bound to MSI2 protein. RNA immunoprecipitation and sequencing identified BMX mRNA as the transcript most significantly disrupted from binding to MSI2 protein, after PRKCQ‐AS1 knockdown. PRKCQ‐AS1 or MSI2 knockdown reduces, while its overexpression enhances, BMX mRNA stability and expression, ERK protein phosphorylation and *MYCN* nonamplified neuroblastoma cell proliferation. PRKCQ‐AS1 knockdown significantly suppresses neuroblastoma progression in mice. In human neuroblastoma tissues, high levels of PRKCQ‐AS1 and MSI2 expression correlate with poor patient outcomes, independent of current prognostic markers. AlphaScreen of a compound library identifies NSC617570 as an efficient inhibitor of PRKCQ‐AS1 RNA and MSI2 protein interaction, and NSC617570 reduces BMX expression, ERK protein phosphorylation, neuroblastoma cell proliferation in vitro and tumor progression in mice. The study demonstrates that PRKCQ‐AS1 RNA interacts with MSI2 protein to induce neuroblastoma tumorigenesis, and that targeting PRKCQ‐AS1 and MSI2 interaction with small molecule compounds is an effective anticancer strategy.

## Graphical abstract

PRKCQ‐AS1 is overexpressed due to transcriptional super‐enhancers. PRKCQ‐AS1 RNA forms a complex with MSI2 protein to induce BMX mRNA stabilization and overexpression, ERK protein phosphorylation and neuroblastoma cell proliferation. Compound NSC617570 blocks PRKCQ‐AS1 binding to MSI2, leading to BMX mRNA disassociation from MSI2 protein, BMX reduction, ERK dephosphorylation, neuroblastoma cell proliferation suppression in vitro and tumor growth inhibition in vivo.

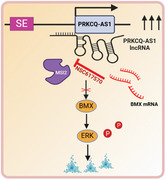

## 1 Introduction

Cancer is the leading cause of disease‐related deaths in children, and neuroblastoma accounts for ≈15% of all childhood cancer deaths. A quarter of human neuroblastoma is driven by *MYCN* oncogene amplification, which has been extensively studied in the past four decades.^[^ ^1^ ^]^ In contrast, *MYCN* nonamplified neuroblastoma without known oncogenic drivers has attracted little research. As their driver oncogenes are unknown, no targeted therapy is available for clinical trials in these patients.

One of the most important drivers of oncogene overexpression is transcriptional super‐enhancers.^[^ ^2^, ^3^, ^4^ ^]^ Transcriptional super‐enhancers are located at the loci of cell identity genes and critical oncogenes and induce massive overexpression of their neighboring genes. The BET bromodomain protein BRD4 and the transcriptional kinase CDK7 substantially augment super‐enhancer‐associated oncogene overexpression. In *MYCN‐*amplified neuroblastoma, *MYCN* transcription is driven by super‐enhancers, BRD4 and CDK7.^[^ ^5^, ^6^, ^7^ ^]^ In addition, long noncoding RNAs (lncRNAs) are emerging as important regulators of oncogene overexpression.^[^ ^8^ ^]^

LncRNAs modulate mRNA and protein expression by regulating gene transcription,^[^ ^9^ ^]^ precursor mRNA splicing,^[^ ^10^ ^]^ mRNA decay and protein translation.^[^ ^8^, ^11^ ^]^ Aberrant lncRNA expression is common in cancer and results in cell differentiation block, proliferation, resistance to cell death, tumor initiation, progression and metastasis.^[^ ^12^, ^13^, ^14^ ^]^ While lncRNAs have been demonstrated to play a critical role in tumorigenesis, there is little literature on therapeutic targeting of oncogenic lncRNAs.

Our RNA sequencing has identified PRKCQ‐AS1 as the lncRNA most overexpressed in *MYCN* nonamplified, compared with *MYCN*‐amplified, human neuroblastoma cell lines. We have found that PRKCQ‐AS1 is overexpressed in *MYCN* nonamplified human neuroblastoma cell lines due to transcriptional super‐enhancers, and that PRKCQ‐AS1 RNA interacts with MSI2 protein to induce BMX mRNA stabilization and overexpression, resulting in neuroblastoma cell proliferation and tumor progression. In addition, we have identified a small molecule compound inhibitor of the PRKCQ‐AS1 RNA and MSI2 protein interaction and have confirmed its anticancer efficacy in vitro and in a mouse model of *MYCN* nonamplified neuroblastoma.

## 2 Results

### 2.1 The lncRNA PRKCQ‐AS1 is Overexpressed in *MYCN* Gene Nonamplified Neuroblastoma Cells

**Figure 1:**
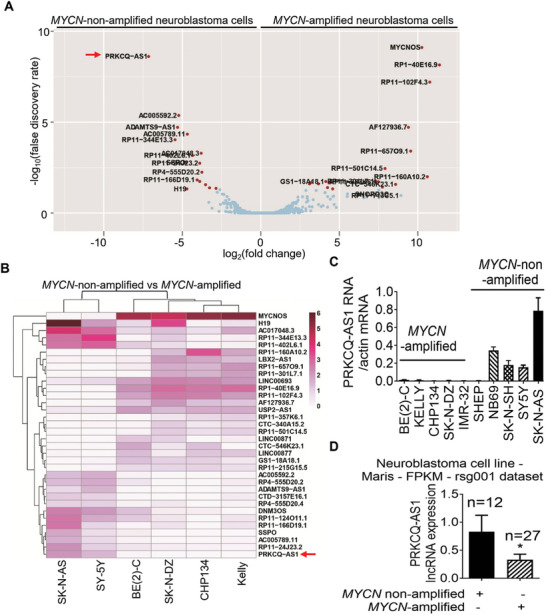
The lncRNA PRKCQ‐AS1 is overexpressed in *MYCN* nonamplified human neuroblastoma cells. A‐B) RNA was extracted from two *MYCN*‐nonamplified (SY5Y and SK‐N‐AS) and four *MYCN*‐amplified (BE(2)‐C, Kelly, CHP134 and SK‐N‐DZ) neuroblastoma cell lines for RNA sequencing analysis of differential gene expression. Volcano plot A) and heatmap B) revealed the top lncRNAs most significantly differentially expressed between *MYCN*‐nonamplified and *MYCN*‐amplified cell lines, with the highest false discovery rate (FDR). C) PRKCQ‐AS1 RNA expression was examined by RT‐PCR with RNA extracted from *MYCN*‐nonamplified and *MYCN*‐amplified human neuroblastoma cell lines. Data were shown as the mean ± standard error of three independent experiments. D) PRKCQ‐AS1 lncRNA expression was extracted from the publicly available Maris – 41 – FPKM – rsg001 RNA sequencing dataset of 39 human neuroblastoma cell lines downloaded from the R2 genomics analysis and visualization platform (http://r2.amc.nl). Differential expression of PRKCQ‐AS1 between *MYCN*‐nonamplified and *MYCN*‐amplified neuroblastoma cell lines was examined by two‐sided unpaired Student's *t*‐test, * indicates *p* < 0.05.

To identify novel lncRNAs important for *MYCN*‐nonamplified neuroblastoma tumorigenesis, we performed RNA sequencing to analyze differential gene expression between two *MYCN* gene nonamplified (SK‐N‐AS, SY5Y) and four *MYCN* gene‐amplified (CHP134, SKNDZ, Kelly and BE(2)‐C) human neuroblastoma cell lines. Differential gene expression analysis of lncRNAs revealed PRKCQ‐AS1 as the top lncRNA overexpressed in the *MYCN*‐nonamplified neuroblastoma cells (log_2_Fold change = −7.13, *p* value = 4.15e^−12^, false discovery rate = 2.40e^−09^) (**Figure** **1**A,B). RT‐PCR also confirmed PRKCQ‐AS1 to be highly expressed in *MYCN*‐nonamplified, compared with *MYCN*‐amplified, human neuroblastoma cell lines (Figure 1C).

Next, we validated the above finding with the publicly available Maris – 41 – FPKM – rsg001 RNA sequencing dataset of 39 human neuroblastoma cell lines (http://r2.amc.nl).^[^ ^15^ ^]^ As shown in Figure 1D, PRKCQ‐AS1 was expressed at a significantly higher level in *MYCN*‐nonamplified than *MYCN*‐amplified human neuroblastoma cell lines.

To confirm the nonprotein‐coding potential of *PRKCQ‐AS1*, Phylogenetic Codon Substitution Frequency (PhyloCSF) analysis using University of California Santa Cruz (UCSC) Genome Brower and LNCipedia^[^ ^16^, ^17^ ^]^ were performed. As shown in Figure S1A,B (Supporting Information), negative PhyloCSF scores, zero Lee translation initiation site^[^ ^18^ ^]^ and zero Bazzini small open reading frame (ORF)^[^ ^19^ ^]^ demonstrated that *PRKCQ‐AS1* lacked small open reading frames and thus exhibited no protein‐coding potential. In addition, LNCipedia analysis revealed human *PRKCQ‐AS1* conservation of 60% in mice and 25% in zebrafish for the short isoform of *PRKCQ‐AS1* (the isoform with two exons), while the long isoform of *PRKCQ‐AS1* (the isoform with three exons) exhibited no conservation among species (Figure S1C, Supporting Information). To determine which of the two isoforms of PRKCQ‐AS1 was the dominant isoform, we transfected SK‐N‐AS cell line with scrambled control siRNA, PRKCQ‐AS1 total siRNA which targeted both isoforms of PRKCQ‐AS1 RNA, PRKCQ‐AS1 long siRNA or PRKCQ‐AS1 short siRNA which targeted the long isoform or the short isoform respectively. RT‐PCR data confirmed that PRKCQ‐AS1 short siRNA and PRKCQ‐AS1 total siRNA exhibited similar knockdown of total PRKCQ‐AS1 RNA, with PRKCQ‐AS1 long siRNA exhibiting significantly lower knockdown of total PRKCQ‐AS1 (Figure S1D, Supporting Information).

Taken together, the data suggest that the lncRNA PRKCQ‐AS1 is significantly overexpressed in *MYCN* nonamplified, compared with *MYCN*‐amplified, neuroblastoma cells. Furthermore, the short isoform of PRKCQ‐AS1 is the dominant isoform and is evolutionarily conserved.

### 2.2 PRKCQ‐AS1 is Overexpressed in *MYCN*‐Nonamplified Neuroblastoma Cells Due to Transcriptional Superenhancers

To understand the mechanism by which PRKCQ‐AS1 is overexpressed in *MYCN*‐nonamplified neuroblastoma, we first examined whether there was *PRKCQ‐AS1* copy number variation in human neuroblastoma tissues using the publicly available single nucleotide polymorphisms (SNP) array dataset which were generated by the Therapeutically Applicable Research to Generate Effective Treatments (TARGET) initiative (https://target‐data.nci.nih.gov/, last accessed June 12, 2013). We previously found that MYCN was not amplified in 74% of the 341 tumors for which the data passed quality control.^[^ ^20^ ^]^ Analysis of the SNP dataset revealed that the *PRKCQ‐AS1* was deleted in 5 and gained in 5 of the MYCN‐nonamplified human neuroblastoma tumors (Table S1, Supporting Information), suggesting that *PRKCQ‐AS1* copy number variation is uncommon in neuroblastoma.

**Figure 2:**
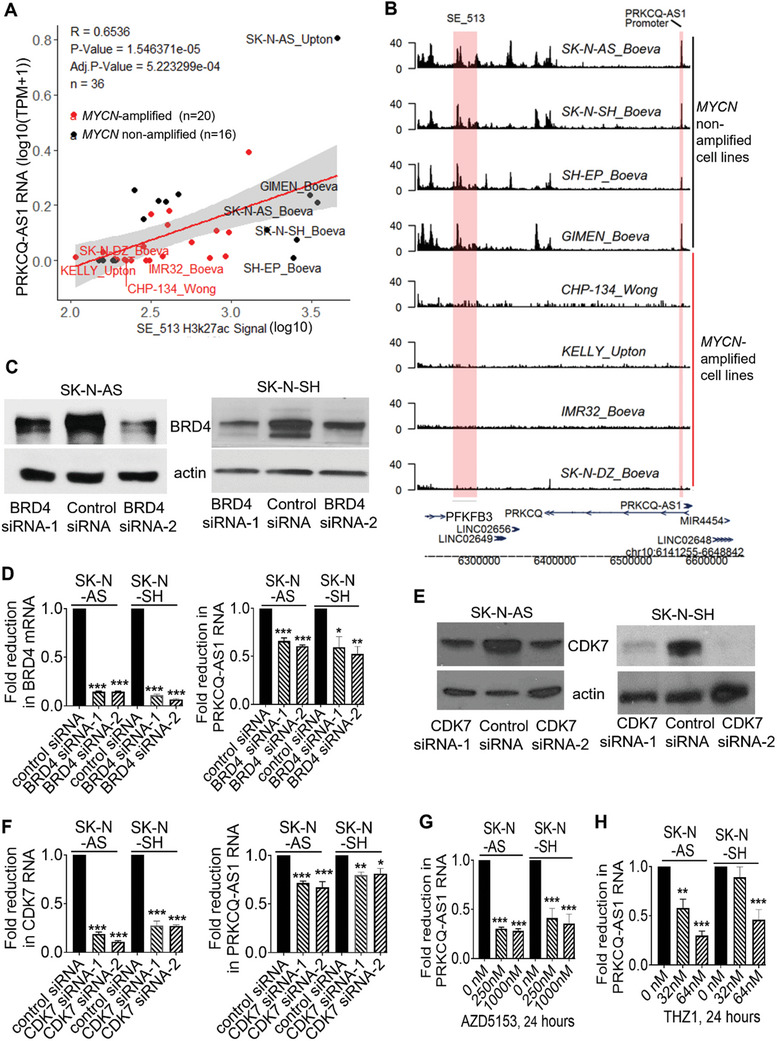
PRKCQ‐AS1 is overexpressed in *MYCN*‐nonamplified neuroblastoma cells due to transcriptional super‐enhancers. A‐B) Publicly available paired H3K27ac ChIP‐Seq and RNA‐Seq data from 36 neuroblastoma cell lines were analyzed. Scatterplot revealed strong positive correlation between cumulative ChIP‐Seq H3K27ac signal at the SE_513 super‐enhancer locus and PRKCQ‐AS1 RNA expression across the 36 neuroblastoma cell lines (A). Track plots showed the SE_513 super‐enhancer in *MYCN* nonamplified SK‐N‐AS, SK‐N‐SH, SH‐EP and GI‐MEN but not *MYCN*‐amplified CHP134, Kelly, IMR32 and SK‐N‐DZ neuroblastoma cell lines at the *PRKCQ‐AS1* gene locus. The *x‐*axis indicated genomic position and the *y*‐axis depicted ChIP‐seq H3K27ac signal, in arbitrary units normalized against input control, and super‐enhancer regions (SE_513) were boxed in red (B). C‐E) SK‐N‐AS and SK‐N‐SH cells were transfected with control siRNA, BRD4 siRNA‐1, BRD4 siRNA‐2 (C‐D), CDK7 siRNA‐1 or CDK7 siRNA‐2 (E) for 48 h. The expression of BRD4, CDK7, and PRKCQ‐AS1 RNA was analyzed by immunoblot and/or RT‐PCR. F‐G) SK‐N‐AS and SK‐N‐SH cells were treated with vehicle control, 250 × 10^−9^ m or 1000 × 10^−9^ m AZD5153 (F), 32 × 10^−9^ m or 64 × 10^−9^ m THZ1 G) for 24 h. RNA was extracted from the cells for RT‐PCR analyses of PRKCQ‐AS1 RNA expression. Data were shown as the mean ± standard error of three independent experiments and evaluated by one‐way ANOVA. *, ** and *** indicates *p <* 0.05, 0.01 and 0.001 respectively.

Super‐enhancers are associated with critical oncogenes and substantially up‐regulate the expression of the associated oncogenes.^[^ ^2^, ^3^, ^4^ ^]^ To examine the cause for PRKCQ‐AS1 overexpression in *MYCN*‐nonamplified neuroblastoma cells, we analyzed published chromatin immunoprecipitation sequencing (ChIP‐Seq) data with antibodies against acetylated histone H3 lysine 27 (acetyl H3K27), the marker for transcriptional super‐enhancers. Publicly available paired H3K27ac ChIP‐Seq and RNA‐Seq data from neuroblastoma cell lines were analyzed to identify super‐enhancers that potentially regulate PRKCQ‐AS1 gene expression (Figure S2A and Table S2, Supporting Information). A strong positive correlation between the cumulative H3K27ac ChIP‐Seq signal at the SE_513 super‐enhancer locus and PRKCQ‐AS1 expression was observed across 36 neuroblastoma cell lines (**Figure** **2**A). Track plots showed the SE_513 super‐enhancer in *MYCN* nonamplified but not *MYCN*‐amplified neuroblastoma cell lines (Figure 2B). We then analyzed the correlation between histone modification protein ChIP‐seq signals at the PRKCQ‐AS1 gene SE_513 super‐enhancer locus and PRKCQ‐AS1 RNA expression using comprehensive paired ChIP‐seq (GSE138315) and RNA‐seq (GSE89413) datasets.^[^ ^15^, ^21^ ^]^ PRKCQ‐AS1 RNA expression showed strong positive correlations with H3K27ac and H3K4me1 signals, no correlation with H3K4me3 (active promoters only) signals, and a strong negative correlation with H3K27me3 (repressed promoters and enhancers) signals at the SE_513 super‐enhancer locus in neuroblastoma cell lines (Figure S2B,C, Supporting Information). As Myc has most recently been reported to directly bind at super‐enhancer loci,^[^ ^22^ ^]^ we observed strong correlations between PRKCQ‐AS1 expression and c‐Myc but not N‐Myc binding at the SE_513 super‐enhancer locus (Figure S2D, Supporting Information). Taken together, the data suggest that PRKCQ‐AS1 is overexpressed in *MYCN*nonamplified neuroblastoma cells due to association with the SE_513 super‐enhancer.

Super‐enhancers regulate associated oncogene transcription through the BET bromodomain protein BRD4^[^ ^2^, ^4^ ^]^ and the transcriptional kinase CDK7.^[^ ^5^, ^23^ ^]^ Our ChIP assays revealed BRD4 protein binding at the SE_513 super‐enhancer (Figure S2E,F, Supporting Information). RT‐PCR and immunoblot analyses showed that transfection with two independent BRD4 siRNAs^[^ ^24^ ^]^ effectively knocked down BRD4 mRNA and protein (Figure 2C,D), and significantly reduced PRKCQ‐AS1 RNA expression (Figure 2D) in SK‐N‐AS and SK‐N‐SH cells. In contrast, BRD4 knockdown did not have similar effects on mRNA expression of PRKCQ, the neighboring sense RNA of PRKCQ‐AS1, and reduced c‐Myc expression in SK‐N‐AS but not SK‐N‐SH cells (Figure S3A,B, Supporting Information). In addition, transfection with two independent CDK7 siRNAs knocked down CDK7 expression and reduced PRKCQ‐AS1 lncRNA expression (Figure 2E,F).

SK‐N‐AS and SK‐N‐SH cells were then treated with the BRD4 inhibitor AZD5153 or the CDK7 inhibitor THZ1. Treatment with AZD5153 or THZ1 reduced neuroblastoma cell proliferation and survival (Figure S3C,D, Supporting Information) and reduced PRKCQ‐AS1 expression (Figure 2G,H). AZD5153 but not THZ1 reduced PRKCQ expression in SK‐N‐AS but not SK‐N‐SH cells, and reduced c‐Myc expression in SK‐N‐SH but not SK‐N‐AS cells (Figure S3E–H, Supporting Information).

Taken together, the data suggest that PRKCQ‐AS1 overexpression in *MYCN*‐nonamplified neuroblastoma cells is due to a transcriptional super‐enhancer, and that PRKCQ, in contrast, is not significantly regulated by transcriptional super‐enhancers.

### 2.3 PRKCQ‐AS1 Promotes *MYCN*‐Nonamplified Neuroblastoma Cell Proliferation

**Figure 3:**
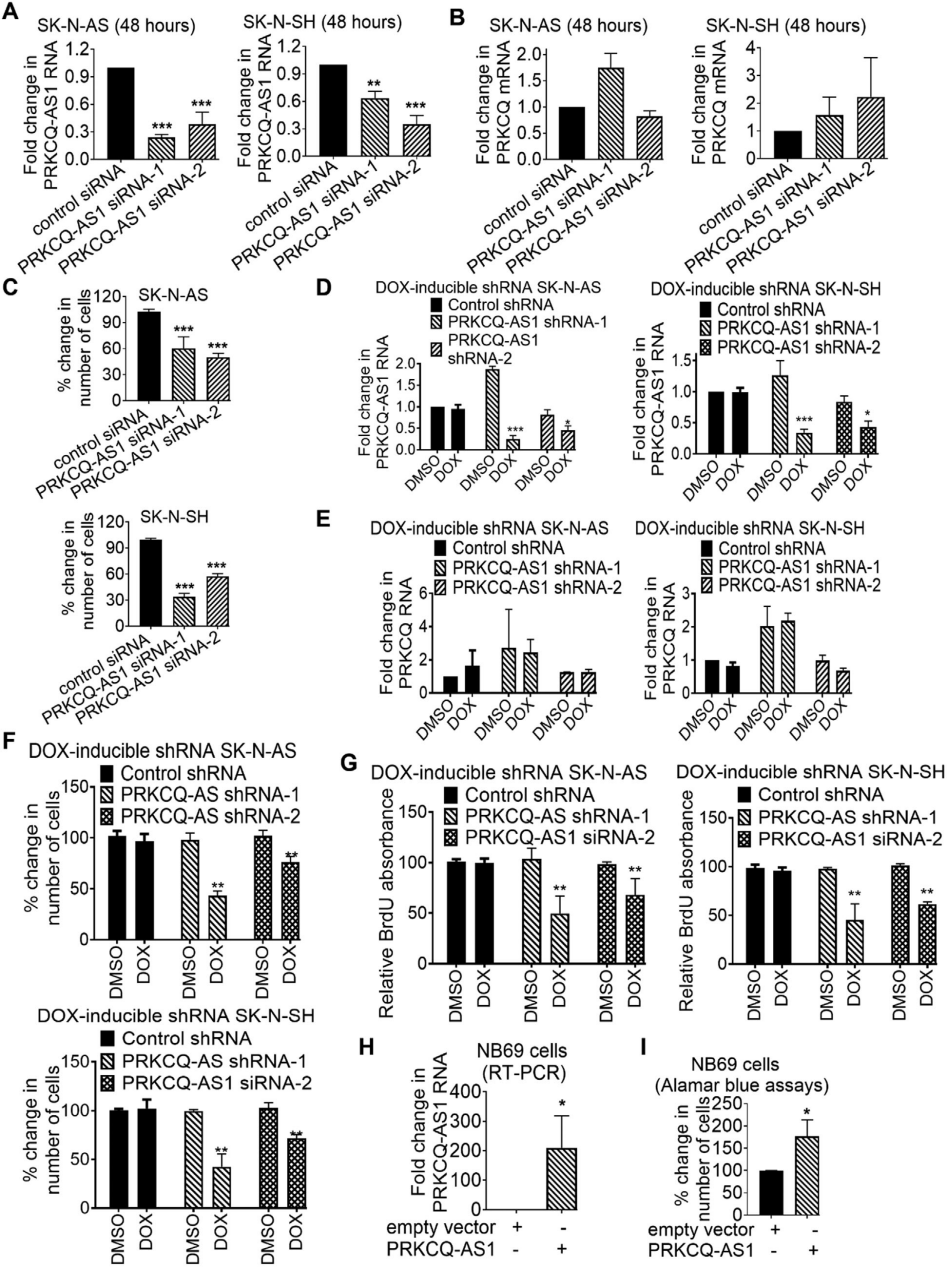
PRKCQ‐AS1 is required for *MYCN*‐nonamplified neuroblastoma cell proliferation. A‐C) SK‐N‐AS and SK‐N‐SH cells were transfected with control siRNA, PRKCQ‐AS1 siRNA‐1 or PRKCQ‐AS1 siRNA‐2. RT‐PCR analysis of PRKCQ‐AS1 (A) and PRKCQ (B) RNA expression was performed 48 h after transfections, and Alamar Blue assays were performed 96 h (SK‐N‐AS cells) or 144 h (SK‐N‐SH cells) after transfections (C). D‐E) DOX‐inducible control shRNA, PRKCQ‐AS1 shRNA‐1 or PRKCQ‐AS1 shRNA‐2 SK‐N‐AS and SK‐N‐SH cells were treated with vehicle control or DOX for 48 h, followed by RT‐PCR analysis of PRKCQ‐AS1 D) or PRKCQ (E) RNA expression. F‐G) DOX‐inducible control shRNA, PRKCQ‐AS1 shRNA‐1 or PRKCQ‐AS1 shRNA‐2 SK‐N‐AS and SK‐N‐SH cells were treated with vehicle control or DOX for 96 h (SK‐N‐AS cells) or 144 h (SK‐N‐SH cells), followed by Alamar blue assays (F) or BrdU incorporation assays (G). H‐I) NB69 *MYCN*‐nonamplified neuroblastoma cells were transfected with an empty vector or PRKCQ‐AS1 expression construct, followed by RT‐PCR analysis of PRKCQ‐AS1 expression (H) and Alamar blue assays (I). Data were shown as the mean ± standard deviation of three independent experiments and evaluated by two‐tailed unpaired Student's *t*‐test for two groups or one‐way ANOVA for more than two groups. * and ** indicated *p* < 0.05 and 0.01 respectively.

As super‐enhancers are exclusively associated with critical oncogenes and cell identity genes,^[^ ^2^, ^4^ ^]^ we examined whether PRKCQ‐AS1 promoted *MYCN*‐nonamplified neuroblastoma cell proliferation. Two independent PRKCQ‐AS1 siRNAs, siRNA‐1 and siRNA‐2, were designed to target different exons of PRKCQ‐AS1 RNA and were transfected into SK‐N‐AS and SK‐N‐SH cells. RT‐PCR analysis confirmed that both PRKCQ‐AS1 siRNA‐1 and siRNA‐2 effectively knocked down PRKCQ‐AS1 RNA expression (**Figure** **3**A) but showed no effect on PRKCQ RNA expression (Figure 3B). Alamar blue assays showed that PRKCQ‐AS1 knockdown reduced neuroblastoma cell proliferation (Figure 3C).

We then established SK‐N‐AS and SK‐N‐SH cells stably transfected with doxycycline (DOX)‐inducible control shRNA, PRKCQ‐AS1 shRNA‐1 or PRKCQ‐AS1 shRNA‐2. The target sequences of the shRNAs were different from the siRNAs. Treatment with DOX knocked down PRKCQ‐AS1 RNA expression (Figure 3D) but had no effect on PRKCQ mRNA expression in DOX‐inducible PRKCQ‐AS1 shRNA cells (Figure 3E). Alamar blue assays showed that treatment with DOX reduced the number of DOX‐inducible PRKCQ‐AS1 shRNA‐1 and PRKCQ‐AS1 shRNA‐2 cells, but not control shRNA SK‐N‐AS and SK‐N‐SH cells (Figure 3F). BrdU incorporation assays showed that PRKCQ‐AS1 knockdown by DOX reduced DOX‐inducible PRKCQ‐AS1 shRNA‐1 and PRKCQ‐AS1 shRNA‐2, but not control shRNA, SK‐N‐AS and SK‐N‐SH cell proliferation (Figure 3G). Next, NB69 *MYCN*‐nonamplified neuroblastoma cells were transfected with an empty vector or PRKCQ‐AS1 expression construct. Alamar blue assays showed that PRKCQ‐AS1 overexpression significantly enhanced cell proliferation (Figure 3H,I). Taken together, the data demonstrate that PRKCQ‐AS1 promotes *MYCN*‐nonamplified neuroblastoma cell proliferation.

### 2.4 PRKCQ‐AS1 is Required for *MYCN*‐Nonamplified Neuroblastoma Cell Clonogenicity In Vitro and Tumor Progression in Mice

**Figure 4:**
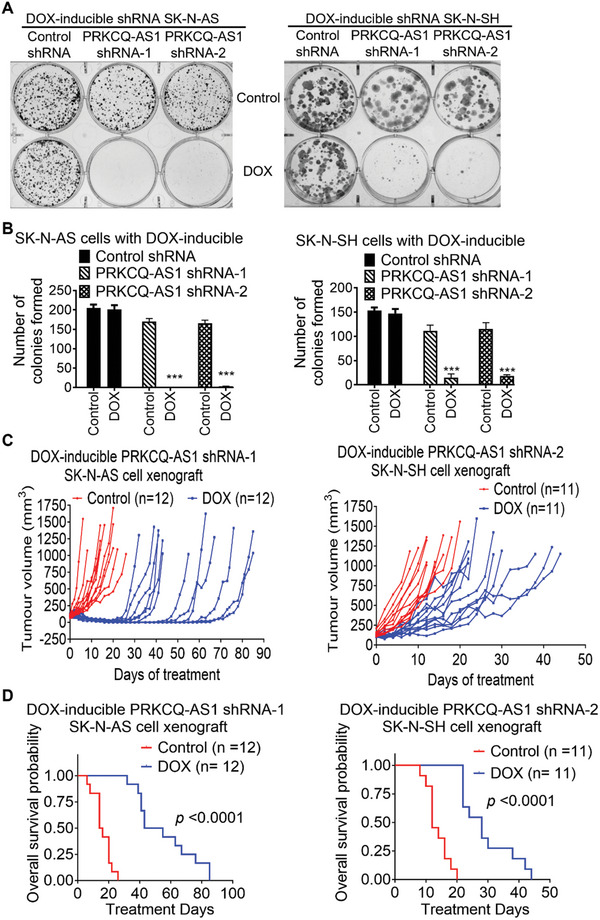
PRKCQ‐AS1 is required for *MYCN*‐nonamplified neuroblastoma cell clonogenicity in vitro and tumor progression in mice. A‐B) DOX‐inducible control shRNA, PRKCQ‐AS1 shRNA‐1 or PRKCQ‐AS1 shRNA‐2 SK‐N‐AS and SK‐N‐SH cells were treated with vehicle control or DOX for 14 days. Cells were then fixed with crystal violet (A) and the numbers of colonies were quantified (B). Data were shown as the mean ± standard error of three independent experiments and evaluated by two‐tailed unpaired Student's *t*‐test. *** indicated *p* < 0.001. C‐D) DOX‐inducible PRKCQ‐AS1 shRNA‐1 SK‐N‐AS and PRKCQ‐AS1 shRNA‐2 SK‐N‐SH cells were xenografted into mice. When tumors reached 0.05cm^3^, the mice were divided into two sub‐groups and fed with food with or without DOX, and tumor growth was monitored. When tumors reached 1.0cm^3^, the mice were culled (C). The probability of overall survival of the mice was analyzed by Kaplan‐Meier survival curves (D). *P* value was obtained from two‐sided log‐rank test.

We examined whether PRKCQ‐AS1 was required for *MYCN*‐nonamplified neuroblastoma cell clonogenic capacity in vitro and tumor progression in vivo. DOX‐inducible control shRNA, PRKCQ‐AS1 shRNA‐1 or PRKCQ‐AS1 shRNA‐2 SK‐N‐AS and SK‐N‐SH cells were treated with vehicle control or DOX. Clonogenic assays showed that treatment with DOX did not influence colony formation in DOX‐inducible control shRNA cells, but substantially blocked clonogenic capacity in DOX‐inducible PRKCQ‐AS1 shRNA‐1 and PRKCQ‐AS1 shRNA‐2 SK‐N‐AS and SK‐N‐SH cells (**Figure** **4**A,B).

DOX‐inducible PRKCQ‐AS1 shRNA‐1 SK‐N‐AS and PRKCQ‐AS1 shRNA‐2 SK‐N‐SH cells were then xenografted into mice. When tumors reached 50 mm^3^, the two groups of mice were each divided into two sub‐groups and provided feed with or without DOX. As shown in Figure 4C, treatment with DOX, compared with control, substantially suppressed neuroblastoma tumor progression. In mice with DOX‐inducible PRKCQ‐AS1 shRNA‐1 SK‐N‐AS cell xenografts, tumor shrank from the third day after DOX treatment and completely regressed in 8 out of 12 mice with the longest tumor‐free days of 41 days. However, tumors re‐grew in all mice after 4–41 days of tumor regression, with all mice eventually succumbing to the disease. Kaplan‐Meier survival analysis showed that PRKCQ‐AS1 knockdown considerably improved overall survival in mice (Figure 4D). These results therefore demonstrate that PRKCQ‐AS1 plays an essential role in *MYCN*‐nonamplified neuroblastoma tumorigenesis.

### 2.5 PRKCQ‐AS1 and Its Binding Protein MSI2 Upregulate BMX mRNA Expression and ERK Protein Phosphorylation

**Figure 5:**
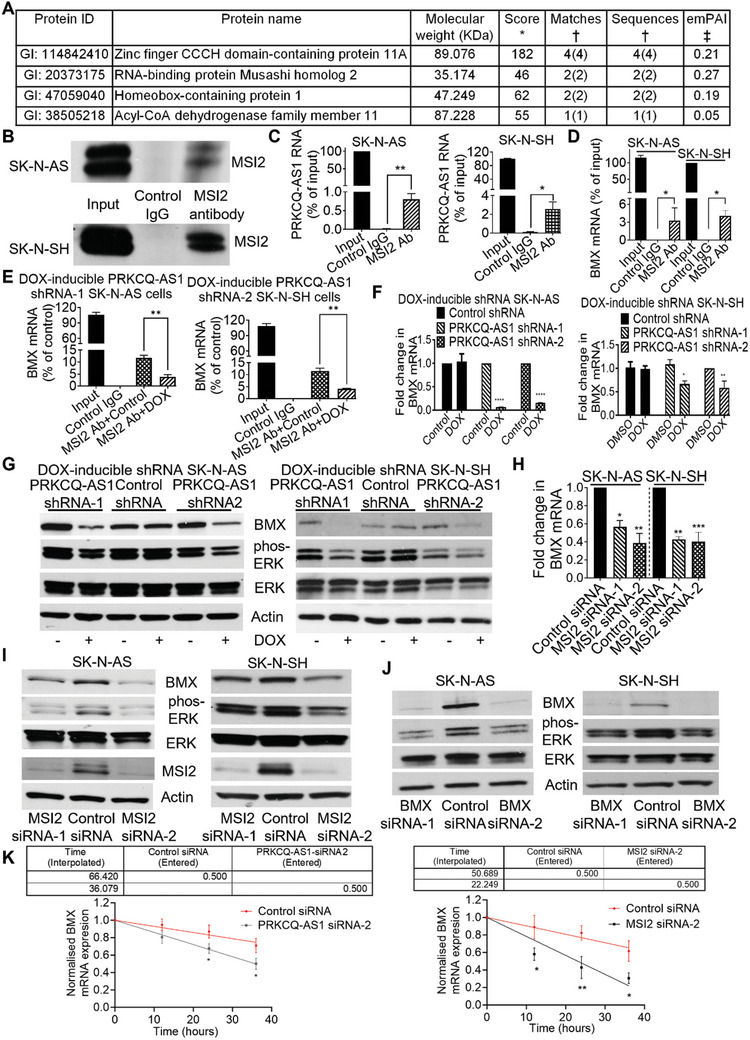
PRKCQ‐AS1 and its binding protein MSI2 up‐regulate BMX mRNA expression and ERK protein phosphorylation. A) PRKCQ‐AS1 RNA was in vitro transcribed and incubated with protein lysates from SK‐N‐AS cells, followed by mass spectrometry analysis of PRKCQ‐AS1 RNA‐binding proteins. *Score: total score from each identified peptide for each protein; †matches/sequences: peptides/sequences for each protein (identified peptides/sequences that matched the corresponding protein); ‡ emPAI: exponentially modified protein abundance index. B‐D) RNA‐immunoprecipitation assays were performed with a control IgG or an anti‐MSI2 antibody (Ab) in SK‐N‐AS and SK‐N‐SH cells. Immunoprecipitated protein was subjected to immunoblot analysis with the anti‐MSI2 Ab (B), and immunoprecipitated RNA was subjected to PCR analysis of PRKCQ‐AS1 RNA (C) and BMX mRNA (D). E) DOX‐inducible PRKCQ‐AS1 shRNA‐1 SK‐N‐AS and PRKCQ‐AS1 shRNA‐2 SK‐N‐SH cells were treated with control or DOX for 48 h, followed by RNA immunoprecipitation assays with a control IgG or an anti‐MSI2 Ab and RT‐PCR analysis of BMX mRNA. F‐G) DOX‐inducible control shRNA, PRKCQ‐AS1 shRNA‐1 and PRKCQ‐AS1 shRNA‐2 SK‐N‐AS and SK‐N‐SH cells were treated with vehicle control or DOX for 48 h, followed by RT‐PCR and immunoblot analyses of BMX mRNA (F) and protein (G) expression, total ERK (ERK) and phosphorylated ERK (phos‐ERK) proteins. H‐I) SK‐N‐AS and SK‐N‐SH cells were transfected with control siRNA, MSI2 siRNA‐1 or MSI2 siRNA‐2 for 48 h, followed by RT‐PCR and immunoblot analyses of BMX mRNA (H) and protein expression and ERK protein phosphorylation (I). J) SK‐N‐AS and SK‐N‐SH cells were transfected with control siRNA, BMX siRNA‐1 or BMX siRNA‐2 for 48 h, followed by immunoblot analyses of BMX expression and ERK phosphorylation. K) SK‐N‐AS cells were transfected with control siRNA, PRKCQ‐AS1 siRNA‐2 or MSI2 siRNA‐2 for 48 h, followed by actinomycin treatment at 5 µg mL^−1^ every 12 h for 36 h. RNA was extracted for RT‐PCR analysis of BMX mRNA expression and half‐life calculation. Data were shown as the mean ± standard error of three independent experiments and evaluated by two‐sided unpaired Student's *t*‐test for two groups or by two‐way ANOVA for more than two groups. *, ** and *** indicated *p* < 0.05, 0.01 and 0.001 respectively.

To identify the mechanism through which PRKCQ‐AS1 exerted tumorigenic effects, we examined the cellular localization of PRKCQ‐AS1 RNA. RT‐PCR analysis showed that PRKCQ‐AS1 RNA is predominantly localized in the cytoplasm in both SK‐N‐AS and SK‐N‐SH cells (Figure S4A,B, Supporting Information). PRKCQ‐AS1 RNA was in vitro transcribed from the pCMV6‐PRKCQ‐AS1 expression construct and biotin‐labeled, then incubated with protein lysates from SK‐N‐AS cells. Mass spectrometry analysis showed that PRKCQ‐AS1 RNA specifically bound to the RNA‐binding protein Musashi homolog 2 (MSI2), zinc finger CCCH domain‐containing protein 11A (ZC11A), homeobox‐containing protein 1 (HMBX1), and acyl‐CoA dehydrogenase family member 11 (ACAD11) proteins (**Figure** **5**A). Among the PRKCQ‐AS1‐binding proteins, MSI2 showed the highest exponentially modified protein abundance index which indicated protein abundance. RNA immunoprecipitation assays showed that a rabbit monoclonal anti‐MSI2 antibody, but not a control rabbit IgG, efficiently pulled down MSI2 protein (Figure 5B) and PRKCQ‐AS1 RNA (Figure 5C) in SK‐N‐AS and SK‐N‐SH cells, demonstrating that MSI2 protein binds to PRKCQ‐AS1 RNA.

Since the identification of lncRNA sequences bound by RNA‐binding proteins is important for deciphering lncRNA function,^[^ ^25^ ^]^ six overlapping fragments (A‐F, < 450 bp for each) of RPKCQ‐AS1 RNA was generated through PCR, biotinylated, incubated with recombinant MSI2 protein, and RNA immunoprecipitated with either an anti‐MSI2 antibody or control rabbit IgG. As shown in Figure S5A, Supporting Information, MSI2 protein bound to both fragment A and fragment B of the PRKCQ‐AS1 RNA, with fragment A exhibiting higher degree of binding. Since there was an overlap between fragment A and fragment B (109 bp overlap) and their total region encompassed 723 base pairs of nucleotides, the 723 nucleotides were further divided into three nonoverlapping fragments (fragments 1–3). RNA immunoprecipitation assays showed that MSI2 protein bound to fragment 1 (65‐306 bp) of the PRKCQ‐AS1 lncRNA (Figure S5B, Supporting Information).

We next investigated the down‐stream targets of the PRKCQ‐AS1 RNA and MSI2 protein interaction. DOX‐inducible control shRNA, PRKCQ‐AS1 shRNA‐1 or PRKCQ‐AS1 shRNA‐2 SK‐N‐AS cells were treated with vehicle control or DOX for 48 h, followed by RNA immunoprecipitation with MSI2 antibody. RNA sequencing analysis of immunoprecipitated RNA showed that BMX mRNA was the RNA most disrupted from binding to MSI2 protein after PRKCQ‐AS1 knockdown (Table S3, Supporting Information). Chromatin immunoprecipitation assays showed that PRKCQ‐AS1 knockdown did not have an effect on RNA polymerase II binding at the BMX gene promoter (Figure S5C,D, Supporting Information), indicating that PRKCQ‐AS1 does not regulate BMX gene transcription. RNA immunoprecipitation assays confirmed that an MSI2 antibody efficiently immunoprecipitated BMX mRNA in SK‐N‐AS and SK‐N‐SH cells (Figure 5D). When BMX mRNA was divided into the coding sequence (CDS), 5′‐untrasnlated region (5′‐UTR) and 3′‐UTR, RNA immunoprecipitation assays showed that MSI2 protein binds to the CDS of BMX mRNA (Figure S5E, Supporting Information). Importantly, immunoprecipitation of BMX mRNA by the MSI2 antibody was significantly reduced, after PRKCQ‐AS1 knockdown by DOX in DOX‐inducible PRKCQ‐AS1 shRNA SK‐N‐AS and SK‐N‐SH cells (Figure 5E). The data demonstrate that PRKCQ‐AS1 is required for MSI2 protein binding to BMX mRNA.

The nonreceptor tyrosine kinase BMX is well‐known to induce ERK protein phosphorylation and thereby induce cancer cell proliferation.^[^ ^26^, ^27^ ^]^ We next examined whether PRKCQ‐AS1 and MSI2 regulated BMX expression and ERK protein phosphorylation. RT‐PCR and immunoblot analyses confirmed that knocking down PRKCQ‐AS1 expression with DOX reduced BMX mRNA and protein expression and reduced ERK protein phosphorylation (Figure 5F,G) but showed no effect on MSI2 expression (Figure S6A, Supporting Information), in SK‐N‐AS and SK‐N‐SH cells. SK‐N‐AS and SK‐N‐SH cells were then transfected with control siRNA or two independent siRNAs targeting different regions of MSI2 RNA, MSI2 siRNA‐1 or MSI2 siRNA‐2. RT‐PCR and immunoblot confirmed that MSI2 siRNAs knocked down MSI2 mRNA (Figure S6B, Supporting Information) and protein expression and reduced BMX mRNA and protein expression and ERK protein phosphorylation (Figure 5H,I), but did not affect PRKCQ‐AS1 RNA expression (Figure S6C, Supporting Information). Consistent with these data, knocking down BMX mRNA (Figure S6D, Supporting Information) and protein expression with two independent BMX siRNAs also reduced ERK protein phosphorylation (Figure 5J).

MSI2 up‐regulates mRNA expression through enhancing their stability. To examine how PRKCQ‐AS1 and MSI2 regulated BMX expression, we performed mRNA stability assays in SK‐N‐AS cells after transfection with control siRNA, PRKCQ‐AS1 siRNA‐2 or MSI2 siRNA‐2. As shown in Figure 5K, PRKCQ‐AS1 or MSI2 knockdown followed by actinomycin D treatment decreased BMX mRNA stability, suggesting that PRKCQ‐AS1 and MSI2 augments BMX mRNA stability. Furthermore, PRKCQ‐AS1 overexpression enhanced BMX mRNA stability, and MSI2 knockdown reversed the effect. Conversely, while MSI2 knockdown alone decreased BMX mRNA stability, forced PRKCQ‐AS1 overexpression significantly blocked the effect (Figure S6E, Supporting Information).

Taken together, the data suggest that PRKCQ‐AS1 RNA binds to MSI2 protein to enhance BMX mRNA stability, upregulate BMX expression and induce ERK protein phosphorylation.

### 2.6 PRKCQ‐AS1 Interacts with MSI2 to Induce Neuroblastoma Cell Proliferation by Upregulating BMX Expression

**Figure 6:**
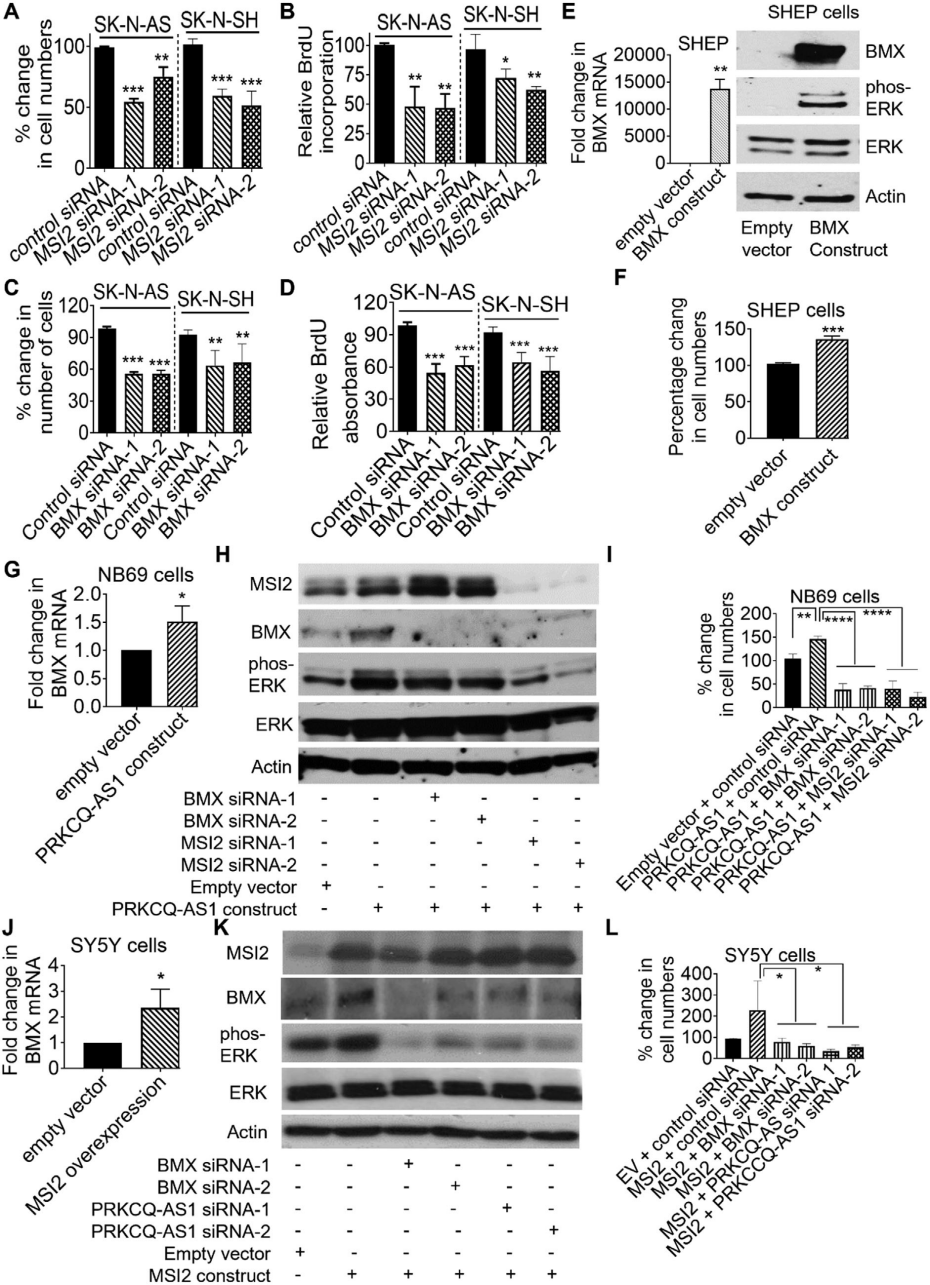
PRKCQ‐AS1 interacts with MSI2 to induce neuroblastoma cell proliferation by up‐regulating BMX expression. A‐D) SK‐N‐AS and SK‐N‐SH cells were transfected with control siRNA, MSI2 siRNA‐1, MSI2 siRNA‐2, BMX siRNA‐1 or BMX siRNA‐2 for 96 h (SK‐N‐AS cells) or 144 h (SK‐N‐SH cells), followed by Alamar blue assays (A, C) or BrdU incorporation assays (B, D). E‐F) SHEP cells were stably transfected with empty vector or BMX expression construct, followed by immunoblot analysis of total ERK (ERK) and phosphorylated ERK (phos‐ERK) proteins 48 h later (E) or Alamar blue assays 72 h later (F). G‐I) NB69 cells were co‐transfected with an empty vector or PRKCQ‐AS1 expression construct, together with control siRNA, BMX siRNA‐1, BMX siRNA‐2, MSI2 siRNA‐1 or MSI2 siRNA‐2, followed by RT‐PCR analysis of BMX mRNA (G), immunoblot analysis of BMX protein expression and ERK protein phosphorylation (H), or Alamar blue assays (I). J‐L) SY5Y cells were co‐transfected with an empty vector or MSI2 expression construct, together with control siRNA, BMX siRNA‐1, BMX siRNA‐2, PRKCQ‐AS1 siRNA‐1 or PRKCQ‐AS1 siRNA‐2, followed by RT‐PCR analysis of BMX mRNA (J), immunoblot analysis of BMX protein expression and ERK protein phosphorylation (K), or Alamar blue assays (L). Data were shown as the mean ± standard deviation of three independent experiments and evaluated by two‐sided unpaired Student's *t*‐test for two groups or by ANOVA for more than two groups. *, ** and *** indicated *p* < 0.05, 0.01 and 0.001 respectively.

We next examined the role of MSI2 and BMX in neuroblastoma cell proliferation. Alamar blue and BrdU incorporation assays showed that MSI2 (**Figure** **6**A,B) or BMX (Figure 6C,D) knockdown with two independent MSI2 siRNAs or BMX siRNAs both reduced SK‐N‐AS and SK‐N‐SH cell proliferation. Consistent with these findings, forced BMX overexpression with an expression construct resulted in increased ERK protein phosphorylation (Figure 6E) and enhanced cell proliferation (Figure 6F) in BMX low expressing *MYCN*‐nonamplified SHEP neuroblastoma cells.

To examine whether MSI2 and BMX are required for PRKCQ‐AS1‐mediated neuroblastoma cell proliferation, we co‐transfected *MYCN*‐nonamplified NB69 neuroblastoma cells with an empty vector or PRKCQ‐AS1 expression construct, together with control siRNA, MSI2 siRNA‐1, MSI2 siRNA‐2, BMX siRNA‐1 or BMX siRNA‐2. RT‐PCR, immunoblot and Alamar blue assays showed that forced PRKCQ‐AS1 overexpression resulted in BMX mRNA and protein up‐regulation, ERK protein phosphorylation and neuroblastoma cell proliferation, and MSI2 or BMX knockdown largely blocked the effects (Figure 6G–I). Conversely, PRKCQ‐AS1 knockdown reduced ERK protein phosphorylation and neuroblastoma cell proliferation, and transfection with an MSI2 or BMX expression construct largely reversed the effects (Figure S7A–D, Supporting Information).

To examine whether PRKCQ‐AS1 and BMX are required for MSI2‐mediated neuroblastoma cell proliferation, we co‐transfected *MYCN*‐nonamplified SY5Y neuroblastoma cells with an empty vector or MSI2 expression construct, together with control siRNA, PRKCQ‐AS1 siRNA‐1, PRKCQ‐AS1 siRNA‐2, BMX siRNA‐1 or BMX siRNA‐2. RT‐PCR, immunoblot and Alamar blue assays showed that forced MSI2 overexpression resulted in BMX mRNA and protein up‐regulation, ERK protein phosphorylation and neuroblastoma cell proliferation, and PRKCQ‐AS1 or BMX knockdown largely blocked the effects (Figure 6J–L).

Taken together, the data suggest that PRKCQ‐AS1 RNA interacts with MSI2 protein to up‐regulate BMX expression and augment ERK protein phosphorylation, leading to neuroblastoma cell proliferation.

### 2.7 High Levels of PRKCQ‐AS1 and MSI2 Expression in Human Neuroblastoma Tissues Independently Predict Poor Patient Prognosis

**Figure 7:**
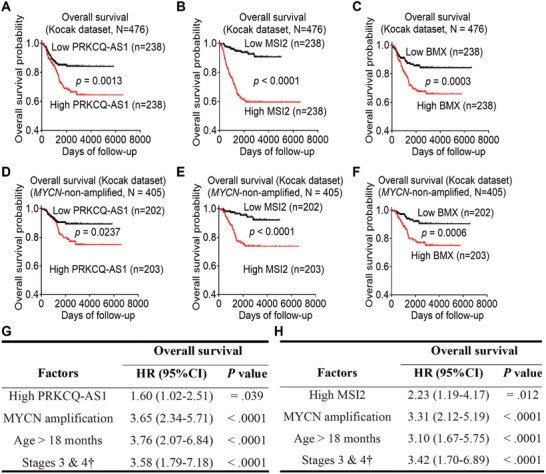
High levels of PRKCQ‐AS1 and MSI2 expression in human neuroblastoma tissues predict poor patient prognosis. A–F) PRKCQ‐AS1, MSI2 and BMX RNA expression in the total cohort of 476 human neuroblastoma tissues (A–C) and the 405 MYCN‐nonamplified human neuroblastoma tissues (D‐F) was extracted from the publicly available microarray gene expression Kocak dataset. Kaplan–Meier curves showed the probability of overall survival of patients according to the median levels of PRKCQ‐AS1 (A and D), MSI2 (B and E) or BMX (C and F) RNA expression. G‐H) Multivariable Cox regression modelling of the Kocak dataset, with PRKCQ‐AS1 (G) and MSI2 (H) expression levels in tumor tissues considered high or low relative to their median expression levels in the 476 neuroblastoma patients of the Kocak dataset. Hazard ratios (HR) were calculated as the antilogs of the regression coefficient in the proportional hazard. The prognostic factors were dichotomized by the median PRKCQ‐AS1 or MSI2 values and tumor stage was categorized as favorable (INSS stages 1, 2, and 4S) or unfavorable (INSS stages 3 and 4). p value was obtained from two‐sided log‐rank test.

To assess the clinical relevance of the above findings, we examined PRKCQ‐AS1, MSI2 and BMX expression in 476 human neuroblastoma tissues in the publicly available neuroblastoma tissue microarray gene expression‐patient prognosis Kocak dataset together with information on patient prognosis, downloaded from the R2 platform (http://r2.amc.nl) (last accessed on July 21, 2020). Using the median RNA expression as the cut‐off, Kaplan‐Meier survival analysis showed that high levels of PRKCQ‐AS1, MSI2 or BMX expression in the total cohort of 476 neuroblastoma tissues (Figure 7A–C), as well as in the 405 *MYCN*‐nonamplified human neuroblastoma tissues (Figure 7D–F), were associated with poor patient prognosis. By contrast, using the median, low or upper quartile of PRKCQ‐AS1, MSI2 and BMX RNA expression as the cut‐off, Kaplan‐Meier survival analyses showed that high levels of PRKCQ‐AS1, MSI2 or BMX expression in *MYCN*‐amplified human neuroblastoma tissues did not correlate with poor patient prognosis (Figure S8A–C, Supporting Information).

Additionally, to determine whether high levels of PRKCQ‐AS1 and MSI2 expression in human neuroblastoma tissues were independent prognostic markers in patients, multivariable COX regression analysis of PRKCQ‐AS1 or MSI2 expression and well‐established neuroblastoma risk factors such as age at the time of diagnosis (> or ≤ 18 months), advanced disease stage (stage 3 and 4) and *MYCN* gene amplification status was performed. When the median expression of PRKCQ‐AS1 and MSI2 was used as the cut‐off point, high levels of PKRCQ‐AS1 or MSI2 expression were associated with poor patient prognosis, independent of age at the time of diagnosis, advanced disease stage and *MYCN* gene amplification status (Figure 7G,H).

Taken together, our data suggests that high levels of PRKCQ‐AS1 and MSI2 expression in tumor tissues independently predict poor prognosis in neuroblastoma patients. (Figure **7**)

### 2.8 The Small Molecule Compound NSC617570 Suppresses the Interaction Between PRKCQ‐AS1 RNA and MSI2 Protein and Shows Anticancer Effects In Vitro and In Vivo

**Figure 8:**
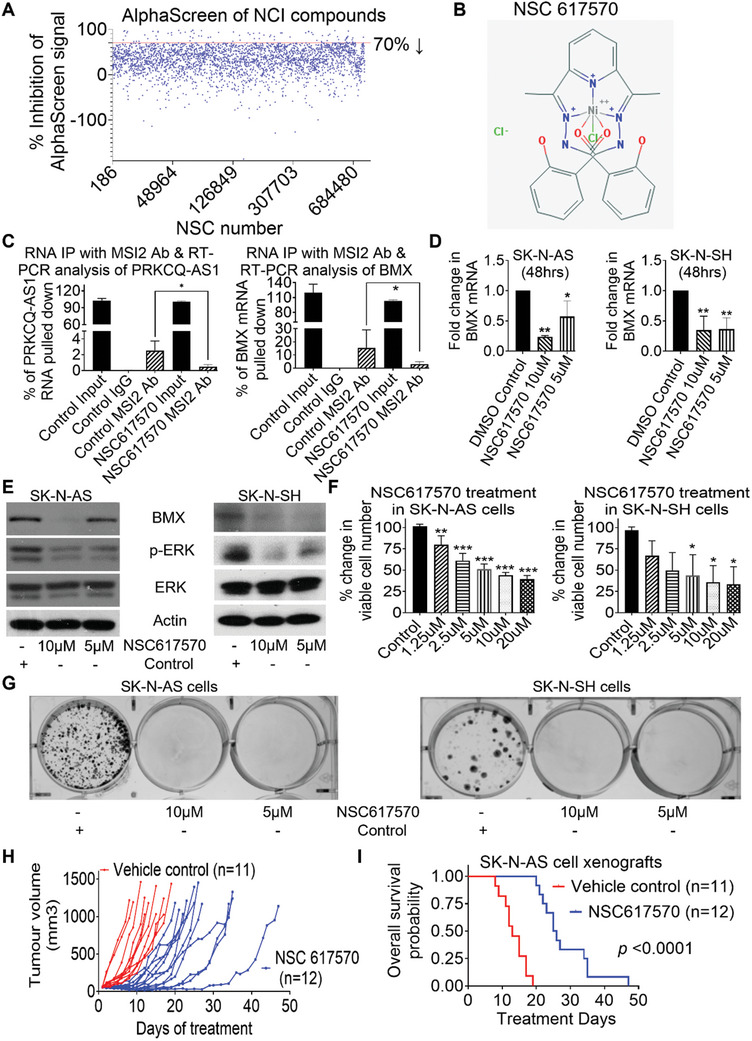
The small molecule compound NSC617570 suppresses the interaction between PRKCQ‐AS1 RNA and MSI2 protein and shows anticancer effects in vitro and in vivo. A) PRKCQ‐AS1 RNA fragment 1 (65‐306 bp at the 5′‐ end) was in vitro transcribed, biotin‐labelled, mixed with Flag‐tagged MSI2 protein, and subjected to AlphaScreen of 2932 small molecule compounds at 10 µM for inhibitors of the interaction between PRKCQ‐AS1 RNA and MSI2 protein. Eight‐five compounds were found to reduce the interaction between PRKCQ‐AS1 RNA and MSI2 protein by > 70% (above the red line). B) Structure of the “hit” compound NSC617570. C) SK‐N‐AS cells were treated with vehicle control or 10 µM NSC617570 for 6 h, followed by RNA immunoprecipitation assays with control IgG or MSI2 antibody and RT‐PCR with primers targeting PRKCQ‐AS1 or BMX. Data were shown as the mean ± standard deviation of three independent experiments and evaluated by Student's *t*‐test. * indicated *p* < 0.05. D‐E) SK‐N‐AS and SK‐N‐SH cells were treated with vehicle control, 5 µM or 10 µM NSC617570 for 48 h, followed by RT‐PCR analysis of BMX mRNA (D) and immunoblot analysis of BMX protein, total ERK protein (ERK) and phosphorylated ERK protein (phos‐ERK) (E). F) SK‐N‐AS and SK‐N‐SH cells were treated with vehicle control or a range of doses of NSC617570 for 72 h, followed by Alamar blue assays. Data were shown as the mean ± standard deviation of three independent experiments and evaluated by ANOVA. *, ** and *** indicated *p* < 0.05, 0.01 and 0.001 respectively. G) SK‐N‐AS and SK‐N‐SH cells were treated with vehicle control, 5 µM or 10 µM NSC617570 for 14 (SK‐N‐AS) or 21 (SK‐N‐SH) days, followed by clonogenic assays. H‐I) SK‐N‐AS cells were xenografted into nude mice. When tumors reached 50 mm^3^, the mice were treated with NSC617570 at 8.75 mg/kg or vehicle control via i.p. injection, 5 times per week. Tumor growth was monitored (H) and mouse overall survival was analyzed (I). For survival analysis, *P* value was obtained from two‐sided log‐rank test.

AlphaScreen technology was originally developed to screen small molecule compound libraries for inhibitors of protein‐protein interaction.^[^ ^28^ ^]^ To discover inhibitors of PRKCQ‐AS1 RNA and MSI2 protein interaction, we in vitro transcribed and biotin‐labelled PRKCQ‐AS1 RNA fragment 1 (65‐306 bp at the 5′‐ end) (Figure S5B, Supporting Information), Flag‐tagged MSI2 protein and performed an AlphaScreen of 2932 small molecule compounds from the National Cancer Institute at 10µM. The primary screening identified 85 compounds which reduced the interaction between PRKCQ‐AS1 RNA and MSI2 protein by > 70% (**Figure** **8**A and Table S4, Supporting Information). A counter screen excluded 69 compounds which nonspecifically interacted with AlphaScreen assay components itself (Table S5, Supporting Information). The remaining 16 compounds were then subjected to secondary screening (Table S6, Supporting Information) across multiple doses, excluding a further 14 compounds due to either a lack of effect or poor structural specificity. Among the two remaining compounds, compound NSC651084 enhanced ERK protein phosphorylation (Figure S9A, Supporting Information) and was excluded; and compound NSC617570 was selected for further studies (Figure 8B).

SK‐N‐AS and SK‐N‐SH cells were treated with vehicle control or NSC617570. RNA immunoprecipitation assays showed that treatment with NSC617570 significantly suppressed MSI2 protein binding to PRKCQ‐AS1 RNA (Figure 8C). Importantly, treatment with NSC617570 also significantly repressed MSI2 protein binding to BMX mRNA (Figure 8C), the target of PRKCQ‐AS1 RNA binding to MSI2 protein. RT‐PCR and immunoblot analyses showed that treatment with NSC617570 decreased BMX mRNA and protein expression and ERK protein phosphorylation (Figure 8D,E), but did not decrease PRKCQ‐AS1 or MSI2 expression (Figure S9B,C, Supporting Information). Alamar blue assays showed that treatment with NSC617570 dose‐dependently reduced neuroblastoma cell proliferation (Figure 8F), and clonogenic assays revealed that treatment with NSC617570 completely blocked neuroblastoma cell colony formation (Figure 8G and Figure S9D, Supporting Information).

We finally examined the anticancer efficacy of NSC617570 in vivo. Maximum‐tolerated dose (MTD) assays identified 8.75 mg/kg via intraperitoneal (i.p.) injection, 5 times per week, as the MTD in mice; while higher doses of NSC617570 at 35, 17.5 or 13.1 mg/kg caused abdominal distention. We then xenografted SK‐N‐AS cells into nude mice. When tumors reached 50 mm^3^, the mice were treated with NSC617570 at 8.75 mg/kg or vehicle control via i.p. injection, 5 times per week. NSC617570 treatment significantly delayed tumor progression and improved mouse survival (Figure 8H,I) but did not cause side effects. Mouse body weight monitoring showed no difference between mice treated with vehicle control and those treated with NSC617570 (Figure S9E, Supporting Information). We have therefore identified NSC617570 as a small molecule compound inhibitor of the interaction between PRKCQ‐AS1 RNA and MSI2 protein and have demonstrated that NSC617570 exerts anticancer effects in vitro and in vivo without significant side effects.

## 3 Discussion

We have recently shown that the lncRNA lncUSMycN and lncNB1 are required for *MYCN*‐amplified neuroblastoma tumorigenesis by inducing N‐Myc mRNA and protein stability respectively.^[^ ^8^, ^20^ ^]^ However, the role of lncRNAs in *MYCN* nonamplified neuroblastoma is poorly understood. In this study, our RNA sequencing data identified PRKCQ‐AS1 as the lncRNA most differentially highly expressed in *MYCN* nonamplified neuroblastoma cell lines. Analysis of published RNA sequencing data from 39 human neuroblastoma cell lines^[^ ^15^ ^]^ confirms that PRKCQ‐AS1 is overexpressed in *MYCN* nonamplified, compared with *MYCN*‐amplified, human neuroblastoma cell lines.

Transcriptional super‐enhancers have been shown to play critical roles in the overexpression of critical oncogenes, such as the *MYCN* oncogene in *MYCN*‐amplified neuroblastoma^[^ ^5^, ^6^ ^]^ and *MYC* oncogene in multiple myeloma and leukemia.^[^ ^2^, ^4^ ^]^ In this study, through analyzing publicly available histone H3K27 acetylation ChIP sequencing datasets, we have identified super‐enhancers at the *PRKCQ‐AS1* gene locus in *MYCN* nonamplified, but not in *MYCN*‐amplified, neuroblastoma cell lines. Pair‐wise comparison of H3K27ac, H3K27me3, H3K4me1 and H3K4me3 signal levels and RNA expression levels in neuroblastoma cell lines reveals positive correlation between H3K27ac and H3K4me1 at the *PRKCQ‐AS1* gene super‐enhancers and PRKCQ‐AS1 RNA expression. In addition, inhibition of the super‐enhancer reader BRD4 or the transcriptional regulator CDK7 significantly reduced the expression of PRKCQ‐AS1, but not MYC and PRKCQ. The data suggests that PRKCQ‐AS1 is overexpressed in *MYCN* nonamplified neuroblastoma cells due to transcriptional super‐enhancers.

In the current study, we have found that PRKCQ‐AS1 knockdown reduces *MYCN* nonamplified neuroblastoma cell proliferation, considerably inhibits clonogenic capacity, suppresses tumor progression and improves survival in two mouse models. In the literature, lncRNAs have been shown to regulate tumorigenesis by interacting with RNA‐binding proteins.^[^ ^8^, ^20^ ^]^ Our RNA‐binding protein pulldown and mass spectrometry analyses identify MSI2 as one of the four PRKCQ‐AS1‐binding proteins. Importantly, while PRKCQ‐AS1 or MSI2 overexpression augments *MYCN* nonamplified neuroblastoma cell proliferation, MSI2 knockdown reverses PRKCQ‐AS1‐induced neuroblastoma cell proliferation, and PRKCQ‐AS1 knockdown blocks MSI2‐mediated neuroblastoma cell proliferation. These data demonstrate that PRKCQ‐AS1 induces tumorigenic effects through interacting with MSI2.

MSI2 induces oncogenic mRNA stabilization or translation, thereby promoting cancer stem cell and cancer cell proliferation and tumorigenesis.^[^ ^29^, ^30^, ^31^, ^32^ ^]^ For example, MSI2 induces prostate cancer cell proliferation by binding to androgen receptor mRNA to enhance its stability and expression.^[^ ^33^ ^]^ By binding to oncogenic Hoxa9, Ikzf2 and Myb mRNAs in leukemia stem cells but not in hematopoietic stem cells and binding to BCAT1 mRNA in leukemia cells, BMX up‐regulate their expression; and suppression of MSI2 induces leukemia cell apoptosis in vitro and inhibits leukemia progression in mice.^[^ ^29^, ^32^, ^34^ ^]^ In this study, our RNA immunoprecipitation sequencing identifies BMX mRNA as the mRNA most disrupted from binding to MSI2 protein after PRKCQ‐AS1 knockdown in neuroblastoma cells. RNA half‐life assays show that PRKCQ‐AS1 or MSI2 knockdown reduces BMX mRNA stability and expression, and PRKCQ‐AS1 overexpression reverses the effect of MSI2 knockdown. While PRKCQ‐AS1 overexpression increases BMX mRNA and protein expression, MSI2 knockdown suppresses the effect. On the other hand, MSI2 overexpression up‐regulates BMX mRNA and protein expression, and PRKCQ‐AS1 knockdown blocks the effect. These data suggest that PRKCQ‐AS1 interacts with MSI2 to up‐regulate BMX mRNA stability and expression.

The nonreceptor tyrosine kinase BMX is preferentially expressed in cancer stem cells and cancer cells. BMX induces ERK protein phosphorylation and activation, glioblastoma cancer stem cell, glioblastoma and prostate cancer cell proliferation and tumorigenesis.^[^ ^35^, ^36^ ^]^ In this study, we have found that BMX knockdown reduces, while BMX overexpression increases, ERK protein phosphorylation and neuroblastoma cell proliferation. Overexpression of PRKCQ‐AS1 or MSI2 enhances BMX expression, ERK protein phosphorylation and neuroblastoma cell proliferation; MSI2 or BMX knockdown blocks the effects of PRKCQ‐AS1 overexpression; and PRKCQ‐AS1 or BMX knockdown blocks the effects of MSI2 overexpression. In human neuroblastoma tissues, high levels of PRKCQ‐AS1, MSI2 or BMX expression predict poor prognosis in *MYCN* nonamplified but not *MYCN*‐amplified patients. Taken together, the data demonstrate that PRKCQ‐AS1 interacts with MSI2 to enhance BMX expression, resulting in ERK protein phosphorylation and *MYCN* nonamplified neuroblastoma tumorigenesis, and that PRKCQ‐AS1 and MSI2 interaction is a valid therapeutic target.

LncRNAs have been shown to be targetable by antisense oligonucleotides and nanoparticles in vitro and in mouse models. However, no antisense oligonucleotide or nanoparticle therapy against oncogenic lncRNAs has been translated into clinical trials. AlphaScreen technology was originally developed to screen small molecule compound libraries for inhibitors of protein‐protein interaction.^[^ ^28^, ^37^ ^]^ In this study, we have modified the AlphaScreen methodology and screened 2932 small molecule compounds from the National Cancer Institute. The natural product NSC617570 has been identified as an efficient inhibitor of PRKCQ‐AS1 RNA and MSI2 protein interaction at µM concentrations. Consistent with data from PRKCQ‐AS1 or MSI2 knockdown, treatment with NSC617570 reduces BMX expression, ERK protein phosphorylation, neuroblastoma cell proliferation and clonogenic capacity. Importantly, treatment with NSC617570 significantly reduces tumor progression without toxicity to normal tissues in a mouse model of human neuroblastoma. Our data provide a proof‐of‐concept small molecule compound PRKCQ‐AS1 and MSI2 interaction inhibitor for further drug discovery, preclinical testing and potential clinical trials.

In summary, this study identifies PRKCQ‐AS1 as the lncRNA most overexpressed in *MYCN* nonamplified, compared with *MYCN*‐amplified, neuroblastoma cell lines, and transcriptional super‐enhancers as the factor responsible for PRKCQ‐AS1 overexpression. PRKCQ‐AS1 RNA interacts with MSI2 protein, resulting in the stabilization and overexpression of oncogenic mRNAs including BMX, ERK protein phosphorylation and neuroblastoma cell proliferation in vitro and tumor progression in vivo. Importantly, the small molecule compound NSC617570 has been identified as an effective inhibitor of PRKCQ‐AS1 and MSI2 interaction. NSC617570 reduces BMX expression, ERK phosphorylation, neuroblastoma cell proliferation in vitro and tumor progression in vivo. In addition, high levels of PRKCQ‐AS1 and MSI2 expression in human neuroblastoma tissues predict poor patient survival rates, independent of current prognostic markers. Our findings therefore identify PRKCQ‐AS1 and MSI2 as important novel co‐factors for *MYCN* nonamplified neuroblastoma tumorigenesis, and provide novel targets and a novel therapeutic agent for the treatment of *MYCN* nonamplified neuroblastoma.

## 4 Experimental Section

### Cell Culture

Human neuroblastoma SK‐N‐AS, SK‐N‐SH, NB69, SY5Y and SHEP cells were cultured in Dulbecco's modified Eagle's medium (DMEM) supplemented with 10% fetal calf serum. 293T cells were obtained from the American Type Culture Collection (Manassas, VA) 20 years ago and SK‐N‐AS cells were obtained from the European Collection of Cell Cultures through Sigma in 2010 (Sigma, Sydney, Australia). All cell lines in this study were tested to be mycoplasma free using the MycoAlert Mycoplasma Detection Kit (Lonza, Rockland Inc, ME) and their identity verified by small tandem repeat profiling conducted at Garvan Institute or Cellbank Australia in 2022 or 2023.

### Establishment of Neuroblastoma Cell Lines Stably Transfected with DOX‐Inducible Control shRNA or PRKCQ‐AS1 shRNA Expression Constructs

The lentiviral DOX‐inducible GFP‐IRES‐shRNA FH1tUTG construct from Dr Marco Herold^[^ ^38^ ^]^ was used to generate SK‐N‐AS and SK‐N‐SH neuroblastoma cells stably transfected with control shRNA, PRKCQ‐AS1 shRNA‐1 or PRKCQ‐AS1 shRNA‐2 expressing construct. PRKCQ‐AS1 shRNA targeting GCTAGAAAGGGCTTGTAAACA (shRNA‐1) and GGACAGTGTTACTTAACAAGG (shRNA‐2) were purchased from IDT (Integrated DNA Technologies, Baulkham Hills, NSW, Australia). PRKCQ‐AS1 sense and antisense shRNA oligoes were cloned into the DOX‐inducible GFP‐IRES‐shRNA FH1tUTG construct and the DOX‐inducible GFP‐IRES‐control shRNA, PRKCQ‐AS1 shRNA‐1 or PRKC‐AS1 shRNA‐2 FH1tUTG construct transfected into 293T cells. After 24 h, the viral media was collected and used to infect SK‐N‐AS and SK‐N‐SH cells after the addition of polybrene (Santa Cruz Biotechnology, Santa Cruz, CA) at 8 µg mL^−1^. The viral media was changed three times over 72 h. GFP‐based cell sorting was used to select cells with top 1% GFP expression. The stable cell lines were treated with 2 µg mL^−1^ DOX (Sigma Aldrich, St Louis, MO, catalogue number D9891) or DMSO vehicle control every 24 h to induce shRNA expression.

### siRNA Transfection

Target sequences of siRNAs were: 5′‐CGACCTAGTTGGTGATTATTT‐3′ (siRNA‐1) and 5′‐GGAAGTAGGCTATGATACATT‐3′ (siRNA‐2) (Dharmacon, Lafayette, CO) for PRKCQ‐AS1; 5′‐CCGGCCTGTGAAACCTCCAAA‐3′ (siRNA‐1) and 5′‐AAGAGGACAAGTGCAAGCCTA‐3′ (siRNA‐2) (Qiagen, Hamburg, Germany) for BRD4; 5′‐GGTGGAAGATGCAATGCTG‐3′ (siRNA‐1) (Thermo Fisher Scientific, Waltham, MA) and 5′‐CAGTAGTCACATAGCTTTA‐3′ (siRNA‐2) (Qiagen) for MSI2; 5′‐CGGAACAAAGTTTCCAGTCAA‐3′ (siRNA‐1) and 5′‐ TGGGTTCTTCGTGGACGGGAA‐3′ (siRNA‐2) (Qiagen) for BMX. To account for any potential nonspecific effects of siRNAs, an Allstars negative control siRNA (Qiagen) was used.

Neuroblastoma cells were plated in either T25 flasks for protein extraction, 6 well plates for RNA extraction or 96 well plates for Alamar blue assay/BrdU assay using Lipofectamine 2000 (Invitrogen). After 8 h, the transfection media was removed and replaced with fresh culture media and analysis performed after the desired transfection time frame.

### Plasmid Transfection

pEZ‐Lv224‐PRKCQ‐AS1 expression construct (catalogue number EX‐E2113‐Lv224) and pReceiver‐Lv213‐MSI2 expression construct (NM_138 962.3, catalogue number EX‐T2560‐Lv213) were purchased from GeneCopoeia (Rockville, MD, USA). pLenti‐C‐mGFP‐BMX expression construct (NM_203 281, catalogue number RC202002L2) was purchased from Origene (Origene, Rockville, MD). For establishing neuroblastoma cells stably expressing the plasmids, 293T cells at 70–80% confluency were transfected with the constructs using Lipofectamine 2000 reagent (Life Technologies, Grand Island, NY) according to the manufacturer's protocol. The following day, the media was removed and replaced with transfection mixture containing plasmid (2 µg), Lipofectamine (50 µL), Opti‐MEM (750 µL) and serum free medium (5 mL) in a T25 flask. After 8 h of incubation, the transfection media was replaced with new media containing 10% fetal calf serum. The viral media was filtered and used to transduce the cell line of interest.

### Real Time Reverse Transcription PCR (RT‐PCR)

RNA was extracted from cells using the PureLink RNA Mini kit (Life Technologies) according to the manufacturer's instructions. RNA concentration and quality were quantified using a Nanodrop (ND) 1000 spectrophotometer (Thermo Fisher Scientific) and cDNAs synthesized with Moloney murine leukemia virus reverse transcriptase (Invitrogen) and random hexanucleotide primers. Quantitative RT‐PCR was performed using gene specific primers and Power SYBR Green Master Mix (Invitrogen) as the fluorescent dye in QuantStudio 3 Real‐Time PCR System (Thermo Fisher Scientific). Nonspecific amplifications were not detected with template controls. The sequences of RT‐PCR primers were: 5′‐ AAGGTGGGACTGCTTTCAAC‐3′ (forward) and 5′‐ AACTGAATAGGGCGGCTACC‐3′ (reverse) for PRKCQ‐AS1; 5′‐ GGATTTTTTTCGGGTAGTGGAA‐3′ (forward) and 5′‐ TTCCTGTTGGTGAAGCTAACGTT‐3′ (reverse) for c‐Myc; 5′‐ AACCATGTCGCCATTTCTTC‐3′ (forward) and 5′‐ TGTCTTCCCGTTGTTCTTCC‐3′ (reverse) for PRKCQ; 5′‐ ATGGCAGAAGCTCTGGAAAA‐3′ (forward) and 5′‐GTGTTTGGTACCGTGGAAA‐3′ for BRD4; 5′‐ GAGCCGAAGTCAGTGGTTGA‐3′ (forward) and 5′‐ACTTCCCGTCCACGAAGAAC‐3′ (reverse) for BMX; 5′‐ ACCTCACCAGATAGCCTTAGAG‐3′ (forward) and 5′‐ AGCGTTTCGTAGTGGGATCTC‐3′ (reverse) for MSI2; 5′‐ CATGGGGAAGGTGAAGGTC‐3′ (forward) and 5′‐ AACAATATCCACTTTACCAGAGTT‐3′ (reverse) for GAPDH; 5′‐ GACGGAGGTTGAGATGAAGC‐3′ (forward) and 5′‐ATTCGGGGCTCTGTAGTCC ‐3′ for MALAT1; 5′‐AGGCCAACCGCGAGAAG‐3′ (forward) and 5′‐ACAGCCTGGATAGCAACGTACA‐3′ (reverse) for Actin. All primers were synthesized by Sigma. Following RT‐PCR, fold changes in target genes relative to the housekeeping gene actin were evaluated using the comparative threshold cycle (△△Ct) method.^[^ ^39^ ^]^

### Immunoblot

Protein was extracted in cold RIPA buffer (150 mM NaCl, 50Mm Tris pH 7.5, 0.5% Sodium deoxycholate, 1% NP‐40 and 0.1% sodium dodecyl sulfate) containing 0.01% 10 × protease inhibitors (Sigma) and phosphatase inhibitors (Roche, Mannheim, Germany). The supernatant was collected after centrifugation at 13000 × g for 20 min at 4 °C. Protein concentration was quantified with the Bicinchoninic Acid Assay kit (Pierce, Rockford, IL). For immunoblot analysis, the protein samples were denatured and loaded onto 12% Criterion precast gels (BioRad, Hercules, CA), followed by electrophoresis and transfer to nitrocellulose membranes. Membranes were blocked with 10% skim milk powder in Tris buffered saline‐tween (TBS‐T) (50 mM Tris‐Cl, pH 7.5, 150 mM NaCl, and 0.1% Tween‐20), and probed with the following primary antibodies: rabbit anti‐Etk/BMX (1:600, CST 24773S, Cell Signaling, Danvers, MA), rabbit anti‐MSI2 (1:1000, Ab76148, Abcam, Cambridge, MA), mouse anti‐p‐p44/42 MAPK (T202/Y204) (phos‐ERK) (1:1000, AB50011, Abcam), rabbit anti‐p44/42 MAPK (ERK) (1:2000, #9102, Cell Signaling) and BRD4 antibody (1:2000, A301‐985A, Bethyl, Montgomery, TA). The membranes were then incubated with a goat anti‐mouse (1:10000, G‐21040, Thermo Fisher Scientific) or goat anti‐rabbit antibody (1:10000, A16116, Thermo Fisher Scientific) conjugated to horseradish peroxidase, and protein bands were visualized with SuperSignal (Pierce). Finally, an anti‐actin antibody (1:10 000, A‐5316, Sigma) was used to probe the membrane as a loading control. All blots were visualized with either X‐ray film or ChemiDoc Imaging System.

### RNA‐Binding Protein Pull‐Down Assays and Mass Spectrometry Analysis

PRKCQ‐AS1 was custom cloned into the pEZ‐Lv224 construct (catalogue number EX‐E2113‐Lv224) by GeneCopoeia. To generate a negative control, the QuikChange II XL site‐directed mutagenesis kit (Aligent, Santa Clara, CA) was used to cut PRKCQ‐AS1 from pEZ‐Lv224 by primer‐based splicing with froward primer 5′‐CGACTCACTATAGGGAGAAACCCAGCTTTCTTGTAC‐3′ and reverse primer 5′‐GTACAAGAAAGCTGGGTTTCTCCCTATAGTGAGTCG‐3′. Two restriction enzyme sites immediately flanking PRKCQ‐AS1 were introduced into the empty vector in the reverse order and PRKCQ‐AS1 ligated to these new sites, generating a negative control. Both vectors were digested, and the linearized product purified using Qiagen PCR purification Kit (Qiagen). In vitro transcription with T7 RNA polymerase (catalogue number 10 881 775 001, Roche) and biotin labelling (Biotin RNA Labelling Mix Kit, catalogue number 11 685 597 910, Roche) of both forward and reverse PRKCQ‐AS1 RNAs were performed according to the manufacturer's protocol. The transcribed forward and reverse PRKCQ‐AS1 RNAs were then purified by Qiagen Mini‐Kit and run on a 1% agarose denaturing gel to confirm RNA transcription at the correct size.

For RNA‐binding protein pulldown assay, ≈2 × 10^7^ SK‐N‐AS cells/sample were harvested by scraping and resuspended in 1.2 mL of lysis buffer [150 × 10^−3^ m NaCl, 50 × 10^−3^ m Tris‐Cl pH 7.5, 0.5% Triton X‐100, 1 × protease inhibitor cocktail and 100 U mL^−1^ of SUPERaseIN (Thermo Fisher Scientific)]. The SK‐N‐AS cell lysate was sonicated using Bioruptor (Diagenode, Liege, Belgium) for 10 min with 30 s on/off cycles and centrifuged for 10 min at 4 °C at the maximum speed. The cell lysate was precleared with 50 µL of activated BcMag Monomer Avidin Magnetic beads (Bioclone, San Diego, CA), according to the manufacturer's protocol. Ethylenediaminetetraacetic acid to a final concentration of 5 mM was added to 20 µg of biotin‐labelled forward and reverse PRKCQ‐AS1 RNAs, followed by the addition of RNA structure buffer to a final concentration of 10 mM Tris pH 7, 0.1 mM KCl and 10 mM MgCl_2_. The mixture was heated to 90 °C for 2 min and cooled at room temperature for 20 min to allow proper secondary structure formation. The biotin‐labelled forward and reverse PRKCQ‐AS1 RNAs were then added to the precleared SK‐N‐AS protein lysate and incubated overnight with rotation at 4 °C. The biotinylated forward and reverse PRKCQ‐AS1 RNA bound proteins were isolated with BcMag Monomer Avidin Magnetic beads according to the manufacturer's protocol and examined by mass spectrometry. Analysis of RNA bound proteins were performed using label‐free quantification in Scaffold 4 and candidate proteins were defined as those that were detectable with a minimum of 2 peptides in all three experimental repeats in SK‐N‐AS cells, with at least 2.5‐fold enrichment compared to the negative control PRKQ‐AS1 RNA pull down.

### RNA‐Immunoprecipitation Assays

Magna RIP Kit from Merck Millipore (Burlington, MA) was used to perform RNA immunoprecipitation assays according to the manufacturer's instructions, with 5 µg of Rabbit Purified IgG antibody (catalogue number PP64B, EZ‐Magna RIP Kit, Merck Millipore) or MSI2 antibody (catalogue number ab76148, Abcam) for immunoprecipitation and primers targeting PRKCQ‐AS1 (5′‐CAAAGGTTCTGCCCTGATGT‐3′ as the forward primer and 5′‐CGGGAGCAGACACATAGGAT‐3′ as the reverse primer), the negative control U1 RNAs or the positive control U1 SNRNP70 for RT‐PCR.

### PRKCQ‐AS1 RNA Fragment Immunoprecipitation

The pEZ‐Lv224‐PRKCQ‐AS1 construct (GeneCopoeia) was used to design PCR primers for PCR to generate 6 overlapping fragments (A – F) of PRKCQ‐AS1 (<450 bp each), with fragment A and B subsequently being divided into 3 nonoverlapping fragments (1 – 3) (primers sequences were listed in Table S7 in the Supporting Information). The amplified PCR products were run on gels and purified using the QIAquick PCR Purification Kit (catalogue number 28 104, Qiagen) according to the manufacturer's protocol. The purified DNA was then in vitro transcribed using the MEGA script kit (AM1333, Thermo Fisher Scientific). The RNA obtained was further purified using the RNeasy Mini Kit (catalogue number 74 104, Qiagen) according to manufacturer's protocol and biotinylated at the 3′ end using the RNA 3′ End Biotinylation Kit (catalogue number 20 160, Pierce). RNA fragment immunoprecipitation was performed in accordance with the steps described in the previous section.

### BMX RNA Fragment Immunoprecipitation

The pLenti‐C‐mGFP‐BMX construct (Origene, RC202002L2) was used to design PCR primers to generate 4 fragments (1–4) of BMX mRNA CDS (primers sequences were listed in Table S8 in the Supporting Information). The amplified PCR products were run on gels and purified using the QIAquick PCR Purification Kit (catalogue number 28 104, Qiagen) according to the manufacturer's protocol. Two in vitro transcription vectors were designed to transcribe the 5′UTR (pT7[mRNA]‐{5′UTR BMX}) and 3′UTR (pT7[mRNA]‐{5′UTR BMX}) (Vector builder). The constructs were transformed in e. coli and Maxi Prep performed (PureLink HiPure Plasmid Filter Maxiprep Kit, catalogue number K210017) according to the manufacturer's protocol, followed by enzymatic digest (SapI) of both plasmids and the products run on agarose gels and purified using the QIAquick PCR Purification Kit (catalogue number 28 104, Qiagen). All purified DNA (both CDS and UTRs) were then in vitro transcribed using the MEGA Script kit (AM1333, Thermo Fisher Scientific). The RNA samples obtained were further purified using the RNeasy Mini Kit (catalogue number 74 104, Qiagen) according to manufacturer's protocol and biotinylated at the 3′ end using the RNA 3′ End Biotinylation Kit (catalogue number 20 160, Pierce). RNA fragment immunoprecipitation was performed in accordance with the steps described in the previous section.

### Paired RNA‐seq and ChIP‐seq Data Analysis

Paired‐end RNA‐seq and single‐end H3K27ac, H3K4me1, H3K4me3, H3K27me3, c‐Myc and N‐Myc ChIP‐seq data for 36 cell lines were retrieved from the short‐read archive (SRA) stored under gene expression omnibus (GEO) accessions GSE89413, GSE90683, GSE113998, GSE138315 and GSE113139. Adapter, quality and length trimming of reads were then undertaken using TrimGalore v0.6.6 either in paired‐end or single‐end mode.

For ChIP‐seq data, trimmed reads were aligned to the human genome (hg38) using the BWA v0.7.11 aligner (bwa‐mem).^[^ ^40^ ^]^ Aligned reads were subsequently sorted by genomic position and indexed using samtools v1.1.0 (sort and index).^[^ ^41^ ^]^ Duplicate reads were marked using picard‐tools v2.4.1 (MarkDuplicates). Read densities were then normalized for GC content, library size and copy number before calling peaks using HMCan v1.44 with the default parameter configuration for narrow peak detection.^[^ ^42^ ^]^ bigWig files containing normalized read densities were scaled according to the top 5000 peaks in each sample using LILY as previously described.^[^ ^43^ ^]^ Super‐enhancer identification and ranking were then performed after stitching enhancers/peaks contained within 12 500 bp regions whilst excluding promoter regions (defined as ±2500 bp from the transcription start site) using LILY, an adaption of the ROSE algorithm.^[^ ^4^, ^43^, ^44^ ^]^ Super‐enhancers were then sorted by genomic position using bedops v2.4.35 (sort‐bed).^[^ ^45^ ^]^ A consensus set of super‐enhancer regions across all samples were then established using bedtools v2.29.1 (multiIntersectBed).^[^ ^46^ ^]^ Cumulative read densities at super‐enhancer loci across all cell lines were then calculated using the GenomicRanges v1.36.1 R package (R v3.6.1).^[^ ^47^ ^]^ Track plots were visualised using the trackplot v1.3.05 R package and bwtool v1.0.^[^ ^48^ ^]^ Genomic dot plots were visualised with the karyoploteR v1.10.5 R package.^[^ ^49^ ^]^ Super‐enhancer rank plots were visualized using the ggplot2 v3.3.3 R package.

For RNA‐seq data, trimmed read pairs were aligned to the human genome (hg38) using the splice‐aware aligner STAR v2.7.5c in two‐pass mode.^[^ ^50^ ^]^ Aligned reads were subsequently sorted by genomic position and indexed as above. Aligned reads were then quantified at genomic loci using the HTSeq v0.12.4 python module (htseq‐count) (Python v3.7.0).^[^ ^51^ ^]^ Gene counts were then normalized to transcripts per million (TPM) using a custom R script as previously described.^[^ ^52^ ^]^ Correlative scatter plots were visualized using the ggplot2 R package as above. Pearson correlations were calculated, and resultant p‐values were adjusted by the Benjamini‐Hochberg method using the stats v3.6.1 R package.

### RNA Immunoprecipitation Sequencing and Data Analysis

DOX‐inducible control shRNA, PRKCQ‐AS1 shRNA‐1 and PRKCQ‐AS1 shRNA‐2 SK‐N‐AS cells were treated with vehicle control or DOX for 48 h followed by RNA immunoprecipitation with a MSI2 antibody (Ab76148, Abcam). RNA integrity was confirmed on the Nanodrop (ND) 1000 spectrophotometer (Thermo Fisher Scientific) and prepared using the low input SMARTer Stranded Total RNA pico input mammalian preparation. Sequencing runs were performed using the Illumina NextSeq 500 flowcell and generated ≈25 M reads per sample. STAR (v2.5) two‐pass method with quantmode parameter set to TranscriptomeSAM was used to align the sequencing results to the GRCh38 reference genome.^[^ ^50^ ^]^ RSEM (v1.2.31) was used to calculate the raw gene counts and transcripts per million (TPM) counts.^[^ ^53^ ^]^ Differential expression analysis was performed using the R package edgeR. Genes with a p value < 0.05 & FC < ‐1.5 were considered significantly downregulated and genes with *p* value < 0.05 & FC > 1.5 considered significantly upregulated.

### Chromatin Immunoprecipitation (ChIP) Assays

For examining the effect of PRKCQ‐AS1 on BMX gene transcription, DOX‐inducible PRKCQ‐AS1 shRNA‐1 SK‐N‐AS cells were treated with vehicle control or DOX for 24 h. ChIP assays were performed with a control rabbit IgG (PP64B, Merck Millipore) or rabbit anti‐RNA Polymerase II antibody (ab26721, Abcam), followed by real‐time PCR with primers targeting a negative control region at the chromosome 7 centromere or ‐188 to ‐56 bp upstream of the BMX gene transcription start site. The sequences of primers were: 5′‐GAAACTATGGCTTGGGCAAA‐3′ (forward) and 5′‐GAGGGAAGCCACATCAAAAA‐3′ (reverse) for the negative control region at the chromosome 7 centromere; 5′‐ GAGAGCTGCCTGGGAAAAGT‐3′ (forward) and 5′‐ACAGGAAACCACGCTCAACA‐3′ (reverse) for the BMX gene promoter region.

To examine BRD4 protein binding at the PRKCQ‐AS1 gene SE_513 super‐enhancer, ChIP assays were performed with a control rabbit IgG (PP64B, Merck Millipore) or rabbit anti‐BRD4 antibody (sc‐48772, Santa Cruz Biotechnology), followed by real‐time PCR with primers targeting a negative control region at the chromosome 7 centromere or the SE_513 super‐enhancer. The sequences of primers were: 5′‐GAAACTATGGCTTGGGCAAA‐3′ (forward) and 5′‐GAGGGAAGCCACATCAAAAA‐3′ (reverse) for the negative control region at the chromosome 7 centromere; and 5′‐GCATAGCATCAATCCTGGCC‐3′ (forward) and 5′‐CTGTGGCTCTGTCCTCCTAG‐3′ (reverse) for the SE_513 super‐enhancer.

### Alamar Blue Assays

Alamar blue assays were performed to measure the number of viable cells post‐transfection (transient and stable) or post‐treatment. 22 µL of Alamar blue reagent (Life Technologies) was added to each well containing 200 µL of cellular media at the designated time point and the plates were further incubated for 6–8 h at 37 °C and then read on a VICTOR X Light Luminescence Plate Reader at 570/595 nm (Perkin Elmer, Waltham, MA). Results were calculated according to the optical density absorbance units and expressed as percentage changes in the number of viable cells, relative to control samples.

### BrdU Incorporation Assays

Cell proliferation was evaluated using BrdU labelling from the colorimetric Cell Proliferation ELISA BrdU kit (catalogue number 11 647 229 001, Roche). Neuroblastoma cells were transfected with siRNAs, or treated with vehicle control or DOX in 96 well plates for 96 to 144 h. The cells were then incubated with 5‐bromo‐2′‐deoxyuridine (BrdU) for 24 h at 37 °C, fixed and incubated with peroxidase‐conjugated anti‐BrdU POD antibody. After washing, the peroxidase substrate solution containing tetramethyl‐benzidine (TMB) was added, and the plates read on a microplate reader at 370 nm wavelength (reference wavelength: ≈492 nm). Results were calculated according to the optical density absorbance units and expressed as percentage changes in BrdU incorporation, relative to control samples.

### Colony Formation Assay

DOX‐inducible control shRNA, PRKCQ‐AS1 shRNA‐1 or PRKCQ‐AS1 shRNA‐2 SK‐N‐AS and SK‐N‐SH cells were seeded in a 6‐well plate and treated with vehicle control or DOX for 14 days. After 14 days, the colonies were fixed with methanol for 15 min and stained with 0.2% crystal violet (Sigma) for 20 min. The staining reagent was washed, and the macroscopic colonies counted with Image J software (Rasband WS, ImageJ, National Institutes of Health, Bethesda, Maryland, USA, https://imagej.nih.gov/ij/, 1997–2018) and images of the representative colonies obtained and quantified.

### AlphaScreen Assays of Small Molecule Compounds to Discover PRKCQ‐AS1 RNA and MSI2 Protein Interaction Inhibitors

AlphaScreen assays were performed using purified human recombinant MSI2 protein (TP301003, Origene Technologies, Rockville, MD) and PRKCQ‐AS1 RNA fragment 1 (306 base pairs). Optimal RNA, protein and buffer concentrations were determined using cross‐titration of individual components. RNA concentration ranging from 0.05 pmol to 10 pmol and protein concentration ranging from 0.1 pmol to 0.8 pmol was titrated in AlphaScreen assay buffer: HEPES (1.5 × 10^−3^ m pH 7.5), NaCl (10× 10^−3^ m), MgCl_2_ (2 × 10^−3^ m), GuHCL (10× 10^−3^ m), NP40 (0.01%). Prior to addition to the assay, the RNA was allowed to fold at 37 °C for 15 min. 9.5 µL of assay buffer was added to each well of a white 384‐well AlphaPlate (catalogue number 6 005 350, PerkinElmer, Waltham, MA), followed by addition of 1.5 µL of 2932 small molecule compounds (National Cancer Institute Approved Oncology Drugs, Diversity, Mechanistic and Natural Products Sets) diluted to desired concentration, 1 µL of RNA diluted to a final concentration of 0.06 pmol and 1 µL of protein diluted to a final concentration of 0 .4pmol. All dilutions were done using UltraPure DNase/RNase‐Free Distilled Water (Invitrogen, cat No. 10 977 015). One column each of negative and positive control was included in each plate. The plates were sealed and spun at 1000 rpm and then incubated for 60 min at 37 °C. After an hour, the plate was allowed to equilibrate to room temperature, followed by the addition of AlphaLISA anti‐FLAG Acceptor beads (PerkinElmer Pty Ltd, AL112C) at a final concentration of 20 µg mL^−1^. The plates were resealed, spun, and incubated at room temperature for 60 min. Subsequently, streptavidin donor beads (catalogue number 6760002S, PerkinElmer) were added to the plate at a final concentration of 20 µg mL^−1^, and the plates resealed, spun, and further incubated for 60 min at room temperature. Due to photosensitivity of the beads, all bead addition was performed in a dark fume hood. After 60 min, the plates were spun one more time and read on the EnVision 2104 Multilabel Plate Reader (PerkinElmer) equipped with the AlphaScreen module by the manufacturer (excitation at 680 nm and emission at 570 nm).

### In Vivo Mouse Experiments

Animal experiments were approved by the Animal Care and Ethics Committee of University of New South Wales, Australia, and the animals' care was performed in agreement with institutional guidelines. All mice used in in vivo experiments were purchased from Ozgene. For the investigation of the effect of PRKCQ‐AS1 on neuroblastoma tumor progression, 24 female Balb/c nude mice and 22 female nonobese diabetic/severe combined immunodeficiency (NOD/SCID) gamma mice aged 5 to 6 weeks were anesthetized and were injected subcutaneously in the right flank with 8 × 10^6^ PRKCQ‐AS1 shRNA‐1 SK‐N‐AS or 5 × 10^6^ PRKCQ‐AS1 shRNA‐2 SK‐N‐SH stable cells respectively. When tumors reached 0.05 cm^3^ in volume, the mice were divided into two subgroups and fed with feed with or without DOX at 600mg k^−1^g (Meat Free Rat and Mouse ± 600 mg Doxycycline per kg Finished Diet, Specialty Feeds Pty Ltd, Glen Forrest, WA, Australia). Tumor growth was monitored, and tumor volume calculated using (length × width × height)/2. When the tumors reached 1.0 cm^3^, the mice were culled.

To examine the anticancer efficacy of NSC617570 in vivo, we first performed maximum tolerated dose (MTD) study in female Balb/c nude mice. Mice were treated with NSC617570 at 35, 17.5, 13.1, 8.75, and 4.375 mg k^−1^g respectively in 2% HPMC (Hydroxypropyl methylcellulose) +1% Tween+10% DMSO in sterile water via i.p. injection, 5 times per week for a maximum of 3 weeks. The mice at the doses of 35, 17.5 or 13.1 mg k^−1^g of NSC617570 exhibited distended abdomen, and these doses were excluded. The mice treated with 8.75mg k^−1^g or 4.375mg k^−1^g of NSC617570 showed no side effects after 21 days of treatment.

SK‐N‐AS neuroblastoma cells were then xenograted into female Balb/c nude mice. When tumors reached 50 mm^3^, the mice were divided into two groups and treated with vehicle control or NSC617570 at 8.75 mg k^−1^g via i.p. injection, 5 times per week. Tumor growth was monitored. When the tumors reached 1.0 cm^3^, the mice were culled.

### Patient Tumor Sample and Cell Line Gene Expression Analysis

PRKCQ‐AS1, MSI2 and BMX RNA expression in human neuroblastoma tissues from 476 patients and clinical information of the patients were extracted from the publicly available Kocak human neuroblastoma tissue microarray gene expression‐patient prognosis dataset,^[^ ^54^, ^55^ ^]^ downloaded from the R2 Genomics Analysis and Visualization Platform(http://r2.amc.nl). PRKCQ‐AS1 expression was also extracted from the publicly available Maris – 41 – FPKM – rsg001 RNA sequencing dataset from 39 neuroblastoma cell lines, also downloaded from the R2 Genomics Analysis and Visualization Platform.

### Statistical Analysis

All experiments were conducted in triplicates and data analyzed with Prism software (GraphPad). The results were presented as mean ± standard error or standard deviation. For analysis of two samples with single variable, Student's *t*‐test was used. For three or more samples with single variable, One‐Way Analysis of Variable (*ANOVA*) test was used. For data with two variables, two‐way *ANOVA* test followed by Bonferroni's Multiple Comparison post‐test was performed. P values of less than 0.05 were considered statistically significant. Overall survival of patients was defined as the time from diagnosis until death or last contact if the patient did not die. Survival analyses were performed according to the Kaplan and Meier method using GraphPad Prism 6.0, and comparisons of survival curves were performed using two‐sided log‐rank tests. Survival probabilities and hazard ratios (HRs) were provided with 95% confidence intervals (CIs). Proportionality was confirmed by visual inspection of the plots of log(2log(S(time))) versus log(time), which were found to remain parallel.^[^ ^20^ ^]^ Statistical tests were two‐sided and s probability value of 0.05 or less was considered statistically significant.

## Conflict of Interest

The authors declare no conflict of interest.

## Author Contributions

S.M., P.Y.L., J.S., G.M., A.D.W., P.V., C.M., J.W., J.C., J.M., M.W., N.B., P.K., and J.L. performed the experiments, collected the data and analyzed the results. L.J., D.S.Z., T.T., P.M., B.B.C, M.E.D, I.S., X.D.Z., and G.M.M. provided conceptual advice. T.L. designed and supervised the study. S.M., J.S., P.V., and T.L. wrote the manuscript with contributions from the co‐authors.

## Ethics Approval Statement

Animal experiments were approved by the Animal Care and Ethics Committee of University of New South Wales, Australia, and the animals' care was performed in agreement with institutional guidelines.

## Patient Consent Statement

Not applicable

## Permission to Reproduce Material From Other Sources

Not applicable

## Clinical Trial Registration

Not applicable

## Supporting information



Supporting Information

Supplemental Table 1

Supplemental Table 2

Supplemental Table 3

Supplemental Table 4

Supplemental Table 5

Supplemental Table 6

Supplemental Table 7

Supplemental Table 8

## Data Availability

The data that support the findings of this study are available from the corresponding author upon reasonable request.
