# Supplementary material for: The Super Enhancer‐Driven Long Noncoding RNA PRKCQ‐AS1 Promotes Neuroblastoma Tumorigenesis by Interacting With MSI2 Protein and Is Targetable by Small Molecule Compounds
Source: Adv Sci (Weinh). 2025 Mar 18;12(18):2412520. doi: 10.1002/advs.202412520 (PMC12079515; doi:10.1002/advs.202412520)
Supplement: Supplementary file 1 — Supporting Information [file ADVS-12-2412520-s003.pdf]

ADVANCED SCIENCE
Open Access

Supporting Information

for *Adv. Sci.*, DOI 10.1002/advs.202412520

The Super Enhancer-Driven Long Noncoding RNA PRKCQ-AS1 Promotes Neuroblastoma Tumorigenesis by Interacting With MSI2 Protein and Is Targetable by Small Molecule Compounds

*Sujanna Mondal, Pei Y. Liu, Janith Seneviratne, Antoine De Weck, Pooja Venkat, Chelsea Mayoh, Jing Wu, Jesper Maag, Jingwei Chen, Matthew Wong, Nenad Bartonicek, Poh Khoo, Lei Jin, Louise E. Ludlow, David S. Ziegler, Toby Trahair, Pieter Mestdagh, Belamy B. Cheung, Jinyan Li, Marcel E. Dinger, Ian Street, Xu D. Zhang, Glenn M. Marshall and Tao Liu**

Supplementary Figures for

The Super Enhancer-driven Long Noncoding RNA PRKCQ-AS1 Promotes Neuroblastoma Tumorigenesis by Interacting with MSI2 Protein and Is Targetable by Small Molecule Compounds

Sujanna Mondal, Pei Y. Liu, Janith Seneviratne, Antoine De Weck, Pooja Venkat, Chelsea Mayoh, Jing Wu, Jesper Maag, Jingwei Chen, Matthew Wong, Nenad Bartonicek, Poh Khoo, Lei Jin, Louise E. Ludlow, David S. Ziegler, Toby Trahair, Pieter Mestdagh, Belamy B. Cheung, Jinyan Li, Marcel E. Dinger, Ian Street, Xu D. Zhang, Glenn M. Marshall, and Tao Liu, *

A

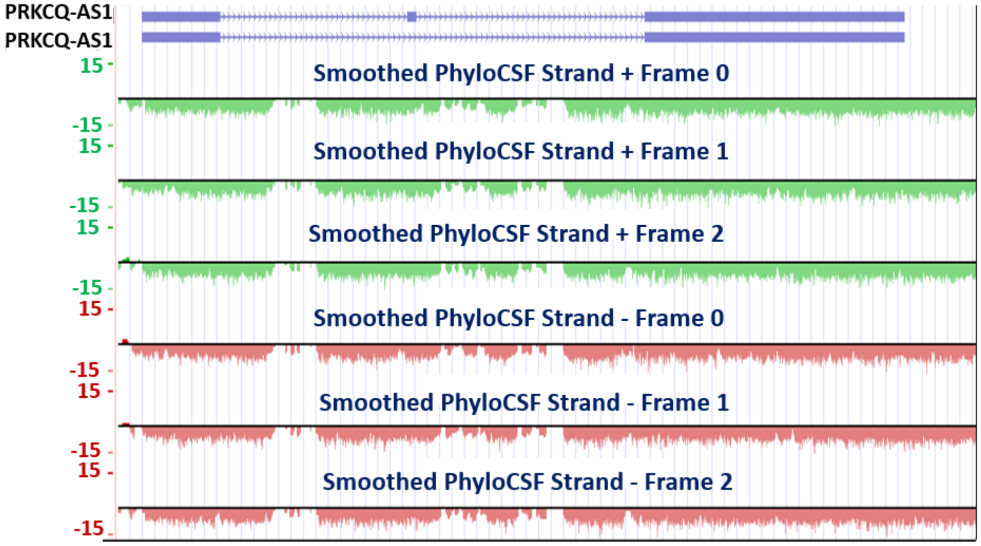

B

	Metric	Raw result	Interpretation
	PRIDE reprocessing 2.0	0	non-coding ?
	Lee translation initiation sites	0	non-coding ?
	PhyloCSF score	-181.5013	non-coding ?
	CPAT coding probability	0.94%	non-coding ?
	Bazzini small ORFs	0	non-coding ?

	Metric	Raw result	Interpretation
	PRIDE reprocessing 2.0	0	non-coding ?
	Lee translation initiation sites	0	non-coding ?
	PhyloCSF score	-181.5013	non-coding ?
	CPAT coding probability	0.79%	non-coding ?
	Bazzini small ORFs	0	non-coding ?

C

Locus conservation	mouse	zebrafish
PRKCQ-AS1 long isoform	no	no
PRKCQ-AS1 short isoform	Yes (60%)	Yes (25%)

D

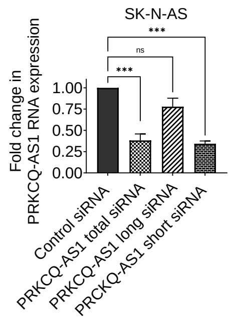

Figure S1. PRKCQ-AS1 exhibits no protein coding potential with the short isoform being evolutionary conserved and the dominant isoform. (A-B) PhyloCSF scores obtained from UCSC Genome Browser **(A)** and LNCipedia **(B)** were used to determine whether PRKCQ-AS1 RNA presented a protein-coding region. In the PhyloCSF score analysis with UCSC Genome Browser, all 6 potential frames were denoted with + or -, and scores below the black line designated that particular frame as non-coding **(A)**. LNCipedia analysis showed negative PhyloCSF scores, low Coding Potential Assessment Tool (CPAT), zero Lee translation initiation site and zero Bazzini small open reading frame (ORF) indicating non-coding **(B)**. **(C)** LNCipedia was used to examine evolutionary signatures that were characteristic of conservation across humans, mice and zebrafish. **(D)** SK-N-AS cells were transfected with scrambled control siRNA, PRKCQ-AS1 total siRNA targeting both isoforms of PRKCQ-AS1 RNA, PRKCQ-AS1 long siRNA or PRKCQ-AS1 short siRNA targeting the long or short isoform respectively, followed by RT-PCR analysis with primers targeting both isoforms of PRKCQ-AS1. Data were shown as the mean $\pm$ standard error of three independent experiments and evaluated by ANOVA. *** indicated $p < 0.001$.

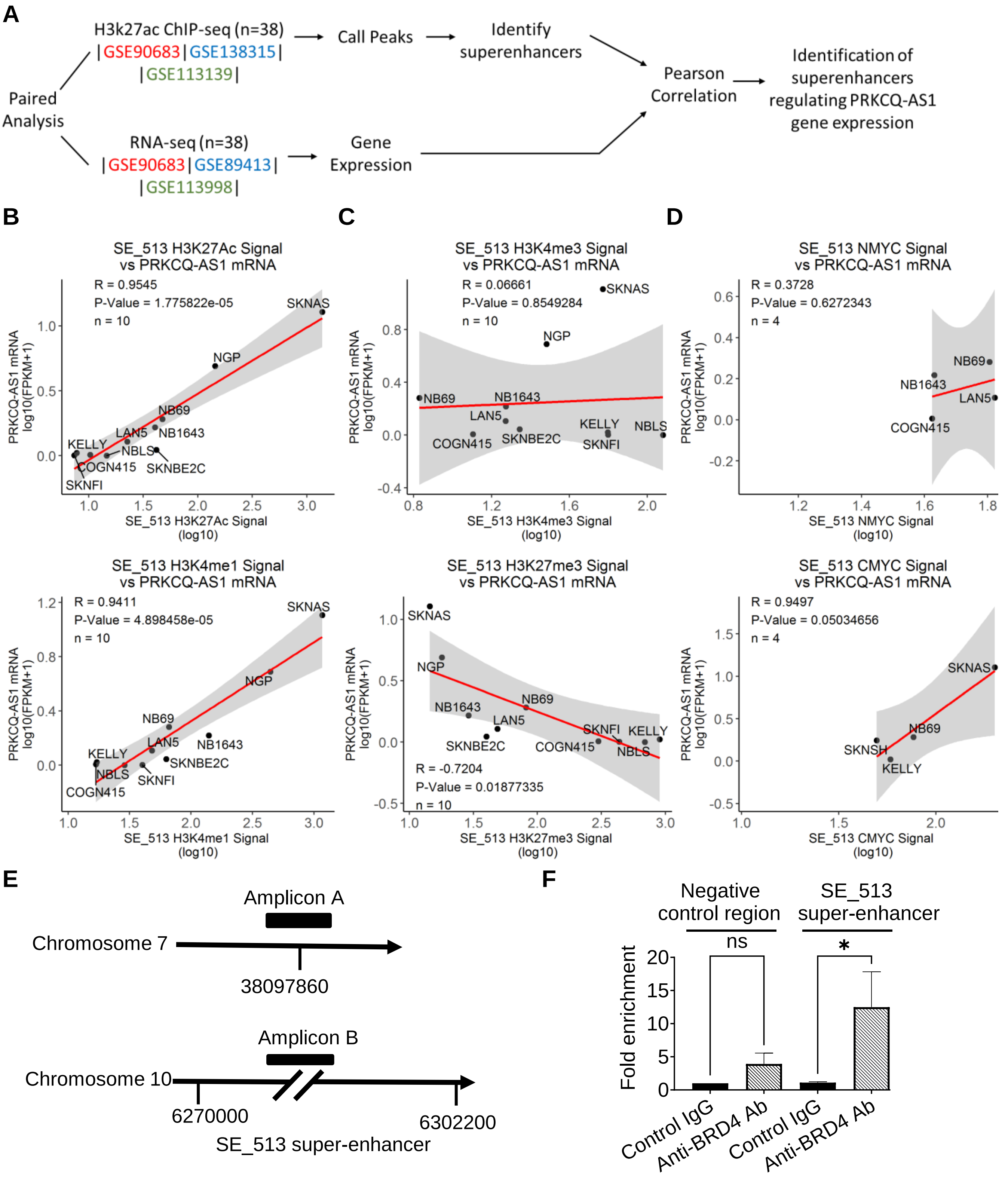
A
H3k27ac ChIP-seq (n=38)
|GSE90683|GSE138315|
|GSE113139|
Call Peaks
Identify superenhancers
Paired Analysis
RNA-seq (n=38)
|GSE90683|GSE89413|
|GSE113998|
Gene Expression
Pearson Correlation
Identification of superenhancers regulating PRKCQ-AS1 gene expression
B
SE_513 H3K27Ac Signal vs PRKCQ-AS1 mRNA
R = 0.9545
P-Value = 1.775822e-05
n = 10
SE_513 H3K27Ac Signal (log10)
PRKCQ-AS1 mRNA log10(FPKM+1)
SE_513 H3K4me1 Signal vs PRKCQ-AS1 mRNA
R = 0.9411
P-Value = 4.898458e-05
n = 10
SE_513 H3K4me1 Signal (log10)
C
SE_513 H3K4me3 Signal vs PRKCQ-AS1 mRNA
R = 0.06661
P-Value = 0.8549284
n = 10
SE_513 H3K4me3 Signal (log10)
SE_513 H3K27me3 Signal vs PRKCQ-AS1 mRNA
R = -0.7204
P-Value = 0.01877335
n = 10
SE_513 H3K27me3 Signal (log10)
D
SE_513 NMYC Signal vs PRKCQ-AS1 mRNA
R = 0.3728
P-Value = 0.6272343
n = 4
SE_513 NMYC Signal (log10)
SE_513 CMYC Signal vs PRKCQ-AS1 mRNA
R = 0.9497
P-Value = 0.05034656
n = 4
SE_513 CMYC Signal (log10)
SKNAS NGP NB69 NB1643 LAN5 KELLY NBLS COGN415 SKNBE2C SKNFI SKNSH
E
Amplicon A
Chromosome 7
38097860
Amplicon B
Chromosome 10
6270000
6302200
SE_513 super-enhancer
F
Negative control region
SE_513 super-enhancer
ns
*
Fold enrichment
Control IgG
Anti-BRD4 Ab
Control IgG
Anti-BRD4 Ab

Figure S2. PRKCQ-AS1 is over-expressed due to association with the SE_513 super-enhancer in *MYCN* non-amplified neuroblastoma cells. (A) Publicly available paired H3K27ac ChIP-seq (GSE90683, GSE138315 and GSE113139) and RNA-seq (GSE90683, GSE89413 and GSE113998) data were processed to call H3K27ac peaks, identify super-enhancers and discern gene expression values for PRKCQ-AS1 which was then correlated with intrachromosomal super-enhancers to identify super-enhancers that regulate PRKCQ-AS1 gene expression. **(B-D)** Scatterplots displaying the relationship between the cumulative H3K27ac, H3K4me1 **(B)**, H3K4me3, H3K27me3 **(C)**, c-Myc and N-Myc **(D)** ChIP-seq signals at the SE_513 super-enhancer loci in the GSE138315 dataset and PRKCQ-AS1 RNA expression in the GSE89413 dataset in up to 10 neuroblastoma cell lines. Correlation statistics were provided in the top or bottom left of the plot, wherein the Pearson correlation coefficient (R), associated raw P-Value and sample size (n) are listed. A linear regression fit was displayed as a red line, with confidence intervals also being displayed in grey. All available neuroblastoma cell lines were annotated. PRKCQ-AS1 expression was presented as Fragments Per Kilobase Million (FPKM) whereas SE_513 signals were presented as arbitrary units following data normalisation and scaling. (**E**) Schematics of the SE_513 super-enhancer region, negative control region and ChIP PCR primer positions. Amplicons A and B indicated the sites for ChIP PCR primers for the negative control region at chromosome 7 centromere and for the SE_513 super-enhancer region at chromosome 10. (**F**) ChIP assays were performed with a control IgG or anti-BRD4 antibody (Ab), followed by PCR with primers targeting the negative control region or the SE_513 super-enhancer region. Error bars represented standard errors from three independent experiments. Data were evaluated by two-way ANOVA, and * indicated $p < 0.05$.

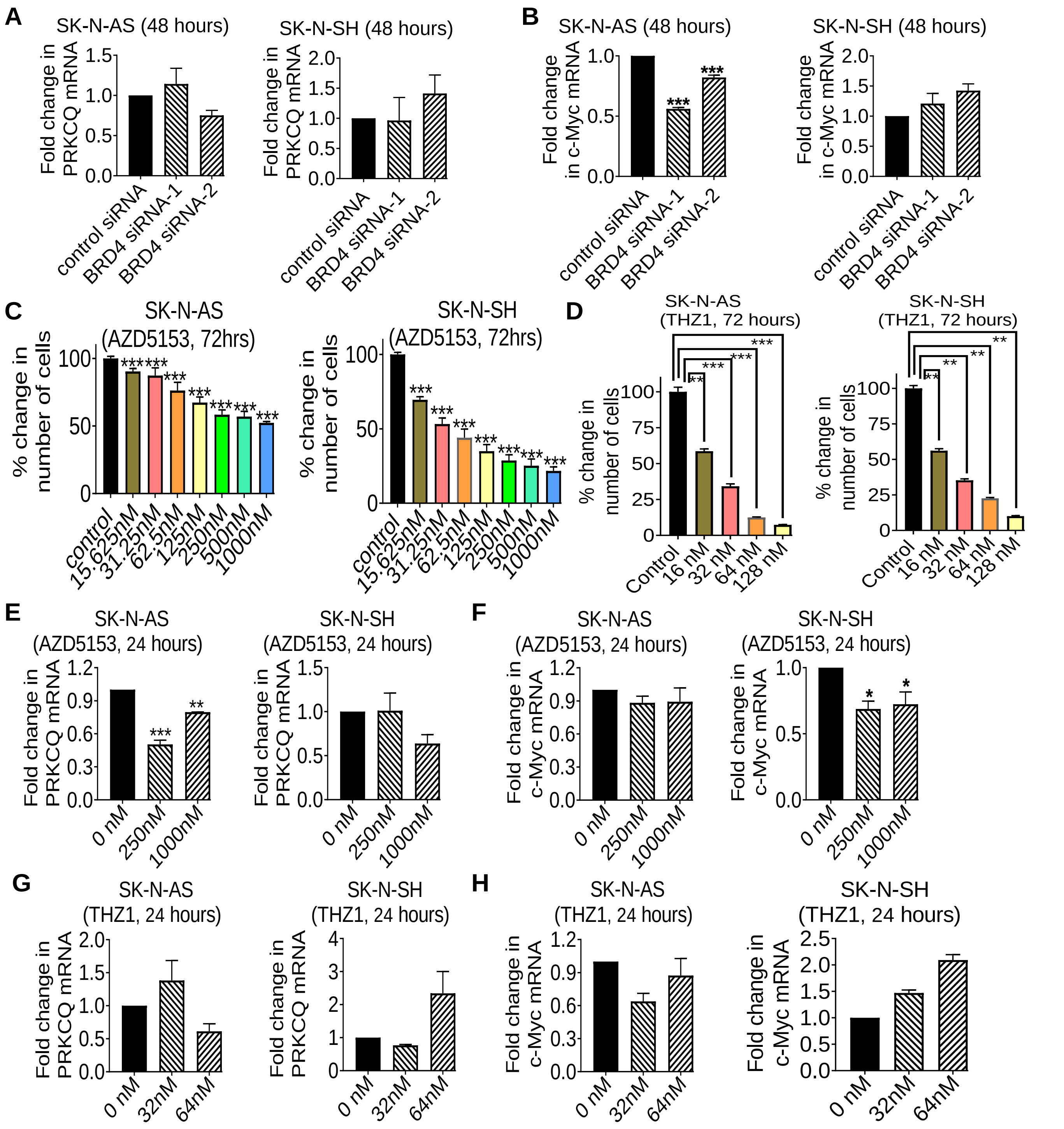
A
SK-N-AS (48 hours)
Fold change in PRKCQ mRNA
control siRNA
BRD4 siRNA-1
BRD4 siRNA-2
SK-N-SH (48 hours)
B
SK-N-AS (48 hours)
Fold change in c-Myc mRNA
SK-N-SH (48 hours)
C
SK-N-AS (AZD5153, 72hrs)
% change in number of cells
control
15.625nM
31.25nM
62.5nM
125nM
250nM
500nM
1000nM
SK-N-SH (AZD5153, 72hrs)
D
SK-N-AS (THZ1, 72 hours)
SK-N-SH (THZ1, 72 hours)
Control
16 nM
32 nM
64 nM
128 nM
E
SK-N-AS (AZD5153, 24 hours)
SK-N-SH (AZD5153, 24 hours)
0 nM
250nM
1000nM
F
SK-N-AS (AZD5153, 24 hours)
SK-N-SH (AZD5153, 24 hours)
G
SK-N-AS (THZ1, 24 hours)
SK-N-SH (THZ1, 24 hours)
32nM
64nM
H
SK-N-AS (THZ1, 24 hours)
SK-N-SH (THZ1, 24 hours)

Figure S3. The expression of PRKCQ and c-Myc is not significantly regulated by transcriptional super-enhancers in *MYCN*-non-amplified neuroblastoma cells. (A-B) SK-N-AS and SK-N-SH cells were transfected with control siRNA, BRD4 siRNA-1 or BRD4 siRNA-2 for 48 hours. The expression of PRKCQ **(A)** and c-Myc **(B)** mRNA was examined by RT-PCR. **(C-D)** SK-N-AS and SK-N-SH cells were treated with various dosages of AZD5153 (0, 15.625, 31.25, 62.5, 125, 250 or 1000 nM) **(C)** or THZ1 (0, 16, 32, 64, 128nM) **(D)** for 72 hours, followed by Alamar blue assays. **(E-H)** SK-N-AS and SK-N-SH cells were treated with vehicle control, 250 or 1000nM AZD5153 **(E, F)**, or 32 or 64nM THZ1 **(G, H)** for 24 hours, followed by RT-PCR analysis of PRKCQ **(E, G)** and c-Myc **(F, H)** mRNA expression. Data were shown as the mean ± standard error of three independent experiments and evaluated by one-way ANOVA. *, ** and *** indicate $p < 0.05$, 0.01 and 0.001 respectively.

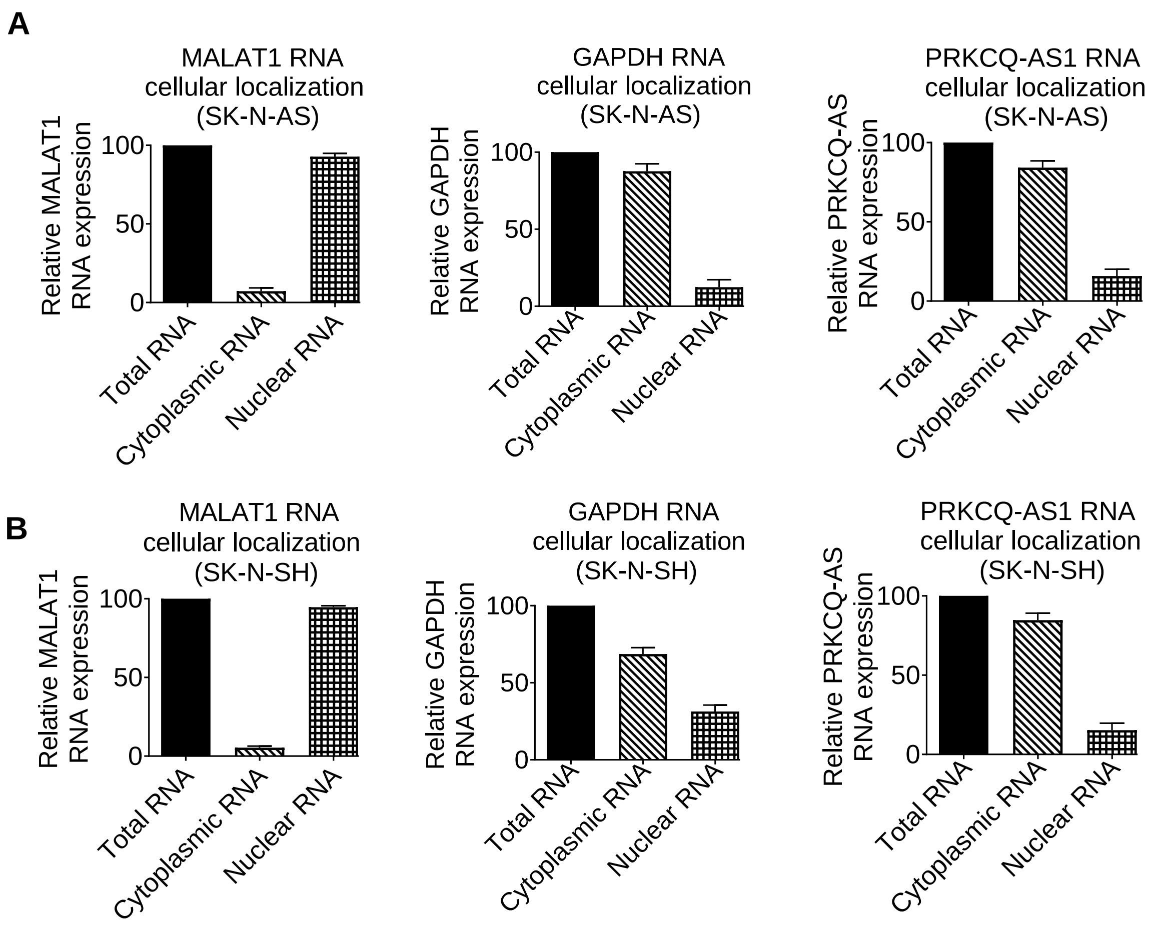

Figure S4. PRKCQ-AS1 RNA is predominantly localized in the cytoplasm. (A-B) RNA was extracted from SK-N-AS **(A)** and SK-N-SH **(B)** cells with or without cytoplasmic and nuclear RNA fractionation. RT-PCR analysis of PRKCQ-AS1 RNA, the nuclear RNA marker MALAT1, and the cytoplasmic RNA marker GAPDH was performed. Error bars represented standard errors from three independent experiments.

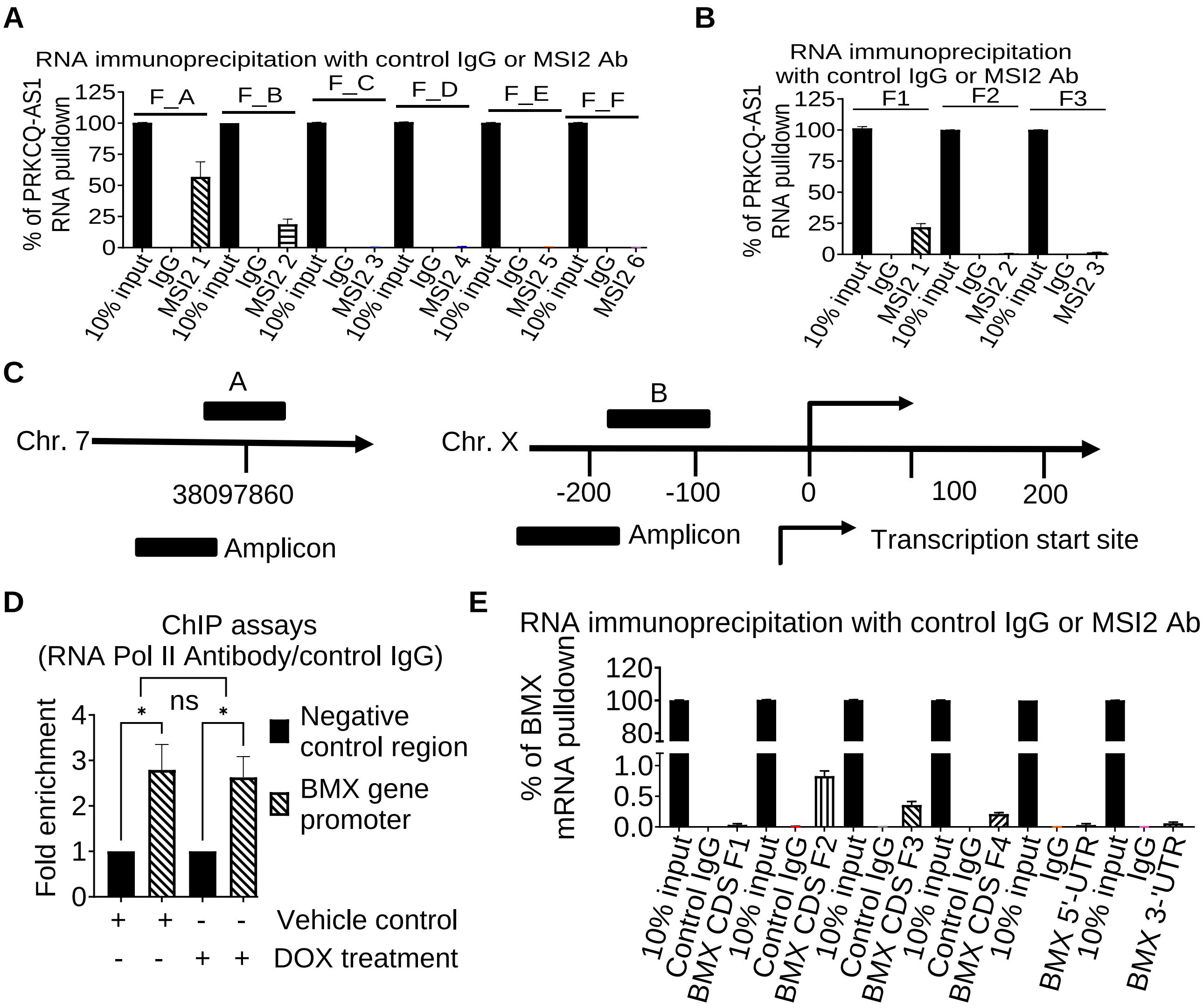
A
RNA immunoprecipitation with control IgG or MSI2 Ab
% of PRKCQ-AS1 RNA pulldown
F_A F_B F_C F_D F_E F_F
10% input IgG MSI2 1 10% input IgG MSI2 2 10% input IgG MSI2 3 10% input IgG MSI2 4 10% input IgG MSI2 5 10% input IgG MSI2 6
B
RNA immunoprecipitation with control IgG or MSI2 Ab
% of PRKCQ-AS1 RNA pulldown
F1 F2 F3
10% input IgG MSI2 1 10% input IgG MSI2 2 10% input IgG MSI2 3
C
A
Chr. 7
38097860
Amplicon
B
Chr. X
-200 -100 0 100 200
Amplicon
Transcription start site
D
ChIP assays
(RNA Pol II Antibody/control IgG)
ns
Fold enrichment
Negative control region
BMX gene promoter
Vehicle control
DOX treatment
E
RNA immunoprecipitation with control IgG or MSI2 Ab
% of BMX mRNA pulldown
10% input Control IgG BMX CDS F1 10% input Control IgG BMX CDS F2 10% input Control IgG BMX CDS F3 10% input Control IgG BMX CDS F4 10% input IgG BMX 5'-UTR 10% input IgG BMX 3'-UTR

Figure S5. MSI2 protein binds to exon 1 of PRKCQ-AS1 lncRNA and the CDS of BMX mRNA. (A) Six overlapping PRKCQ-AS1 RNA fragments (fragments A – F) were incubated with recombinant MSI2 protein, followed by RNA immunoprecipitation assays with an anti-MSI2 antibody (Ab) or control rabbit IgG. The precipitated RNA was examined by RT-PCR analysis of PRKCQ-AS1 RNA fragments. **(B)** Three non-overlapping PRKCQ-AS1 RNA fragments encompassing 723 base pairs at the 5'- end in exon 1 (fragments A and B as shown in (**A**)) were incubated with recombinant MSI2 protein, followed by RNA immunoprecipitation assays with an anti-MSI2 antibody or control rabbit IgG. The immunoprecipitated RNA was examined by RT-PCR analysis of the PRKCQ-AS1 RNA fragments. Error bars represented standard errors from three independent experiments. (**C**) Schematics of the BMX gene and ChIP PCR primer positions. Amplicons A and B indicate the sites for ChIP PCR primers on chromosome (Chr.) 7 centromere (negative control region) and Chr. X. (**D**) DOX-inducible PRKCQ-AS1 shRNA-1 SK-N-AS cells were treated with vehicle control or DOX. ChIP assays were performed with a control IgG or anti-RNA polymerase II (RNA Pol II) antibody and PCR with primers targeting the negative control region at Chr. 7 centromere or the BMX gene promoter. Fold enrichment was calculated as the difference in cycle thresholds obtained with the anti-RNA Pol II antibodies, compared with the control IgG, relative to input. Error bars represented standard errors from three independent experiments. Data were evaluated by two-way ANOVA, and * indicated $p < 0.05$. **(E)** Four overlapping BMX CDS mRNA fragments (BMX CDS F1 – F4), 5'-UTR and 3'-UTR were incubated with recombinant MSI2 protein, followed by RNA immunoprecipitation assays with an anti-MSI2 antibody (Ab) or control rabbit IgG. The precipitated RNA was examined by RT-PCR analysis of BMX mRNA fragments. Error bars represented standard errors from three independent experiments.

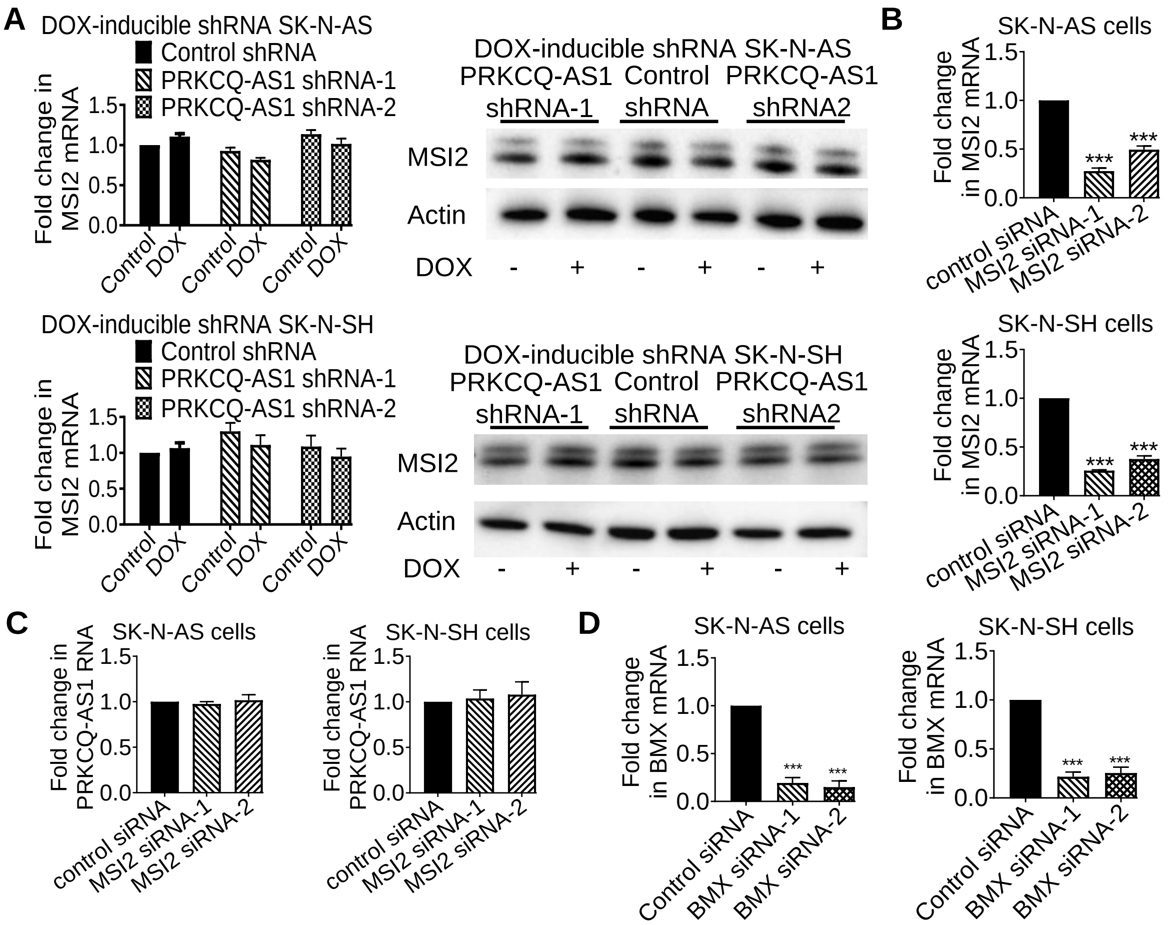

E

Time (Interpolated)	EV + Control siRNA	EV + MSI2 siRNA-2	PRKCQ-AS1 + Control siRNA	PRKCQ-AS1 + MSI2 siRNA-2
37.02339236	0.5			
17.54732103		0.5		
64.68122414			0.5	
27.73332615				0.5

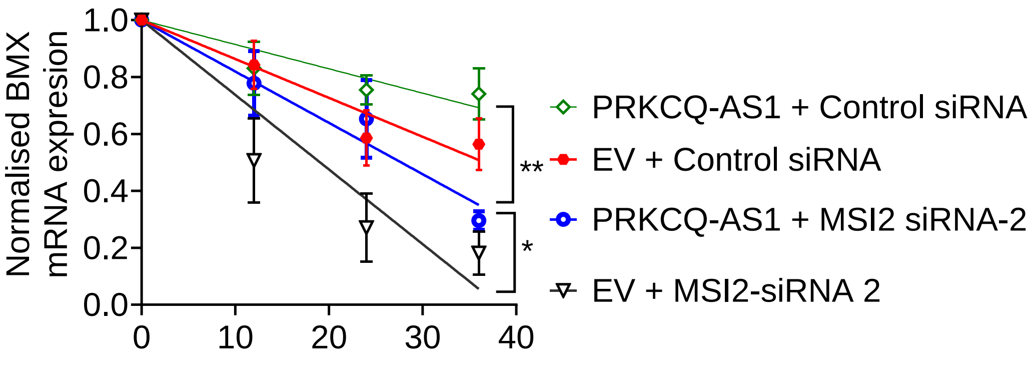

Figure S6. PRKCQ-AS1 and MSI2 do not regulate the expression of each other but interact with each other to stabilize BMX mRNA. **(A)** DOX-inducible control shRNA, PRKCQ-AS1 shRNA-1 or shRNA-2 SK-N-AS and SK-N-SH cells were treated with control or DOX for 48 hours, followed by RT-PCR and immunoblot analyses of MSI2. **(B-D)** SK-N-AS and SK-N-SH cells were transfected with control siRNA, MSI2 siRNA-1, MSI2 siRNA-2 **(B-C)**, BMX siRNA-1 or BMX siRNA-2 **(D)** for 48 hours. RNA was extracted for RT-PCR analysis of MSI2 **(B)**, PRKCQ-AS1 **(C)** or BMX **(D)** RNA expression. Data were shown as the mean ± standard error of three independent experiments and evaluated by one-way ANOVA. *** indicates $p < 0.001$. **(E)** NB69 cells were co-transfected with an empty vector or PRKCQ-AS1 expression construct, together with control siRNA or MSI2 siRNA-2, for 48 hours, followed by actinomycin treatment at 5 μg/ml every 12 hours for 36 hours and RT-PCR analysis of BMX mRNA expression and half-life calculation. Data were shown as the mean ± standard error of three independent experiments and evaluated by two-way ANOVA. * and ** indicated $p < 0.05$ and 0.01 respectively.

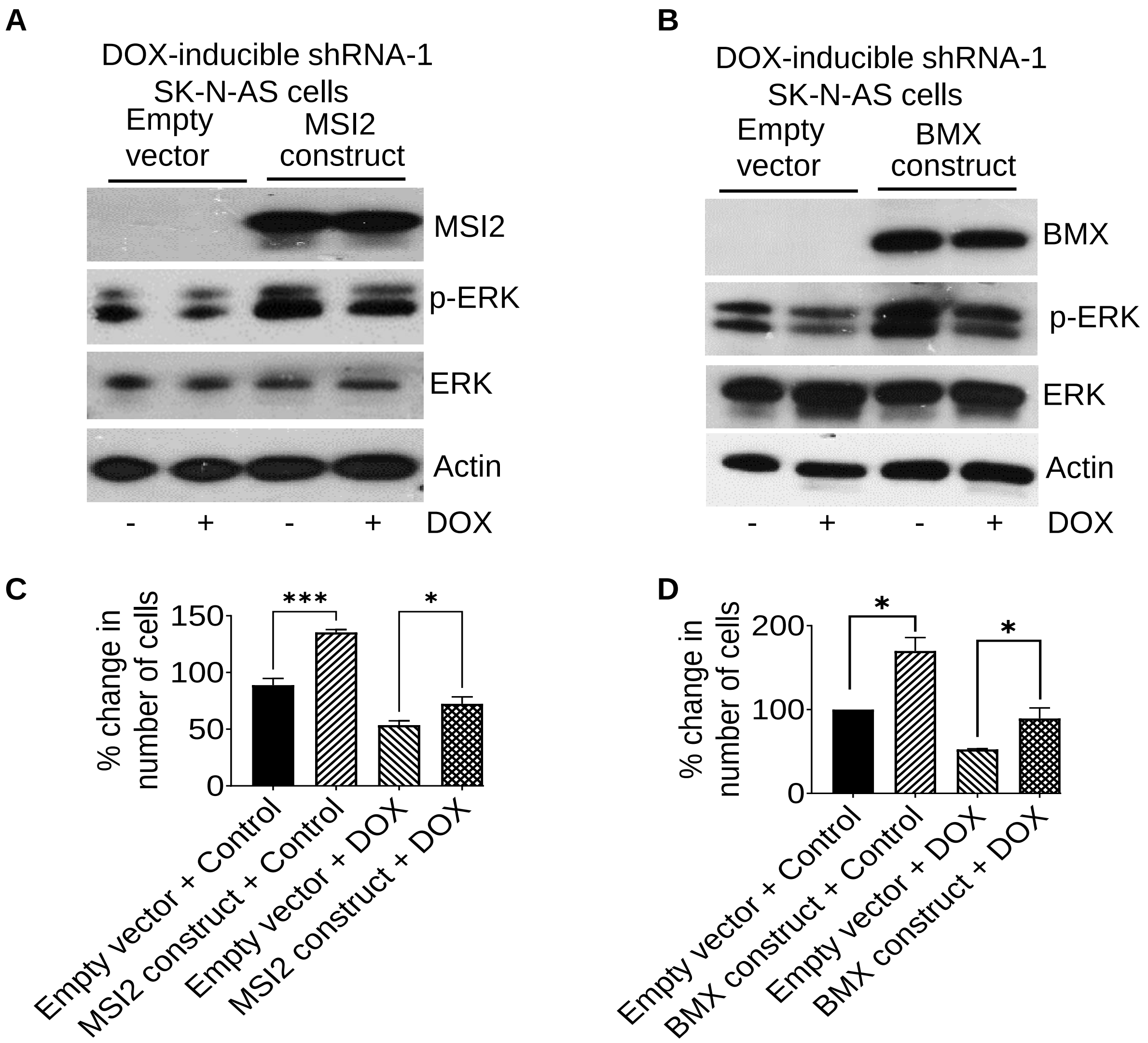

Figure S7. MSI2 and BMX reverse ERK protein de-phosphorylation and neuroblastoma cell growth inhibition due to PRKCQ-AS1 knockdown. (A-D) DOX-inducible control shRNA, PRKCQ-AS1 shRNA-1 SK-N-AS cells were transfected with an empty vector, MSI2 expression construct **(A, C)** or BMX expression construct **(B, D)**, followed by treatment with vehicle control or DOX. Protein was extracted from the cells 48 hours later for immunoblot analysis **(A-B)**, and the cells were subjected to Alamar blue assays 96 hours later **(C-D)**. Data were shown as the mean ± standard error of three independent experiments and evaluated by two-tailed unpaired Student's *t*-test. * and *** indicated $p < 0.05$ and 0.001 respectively.

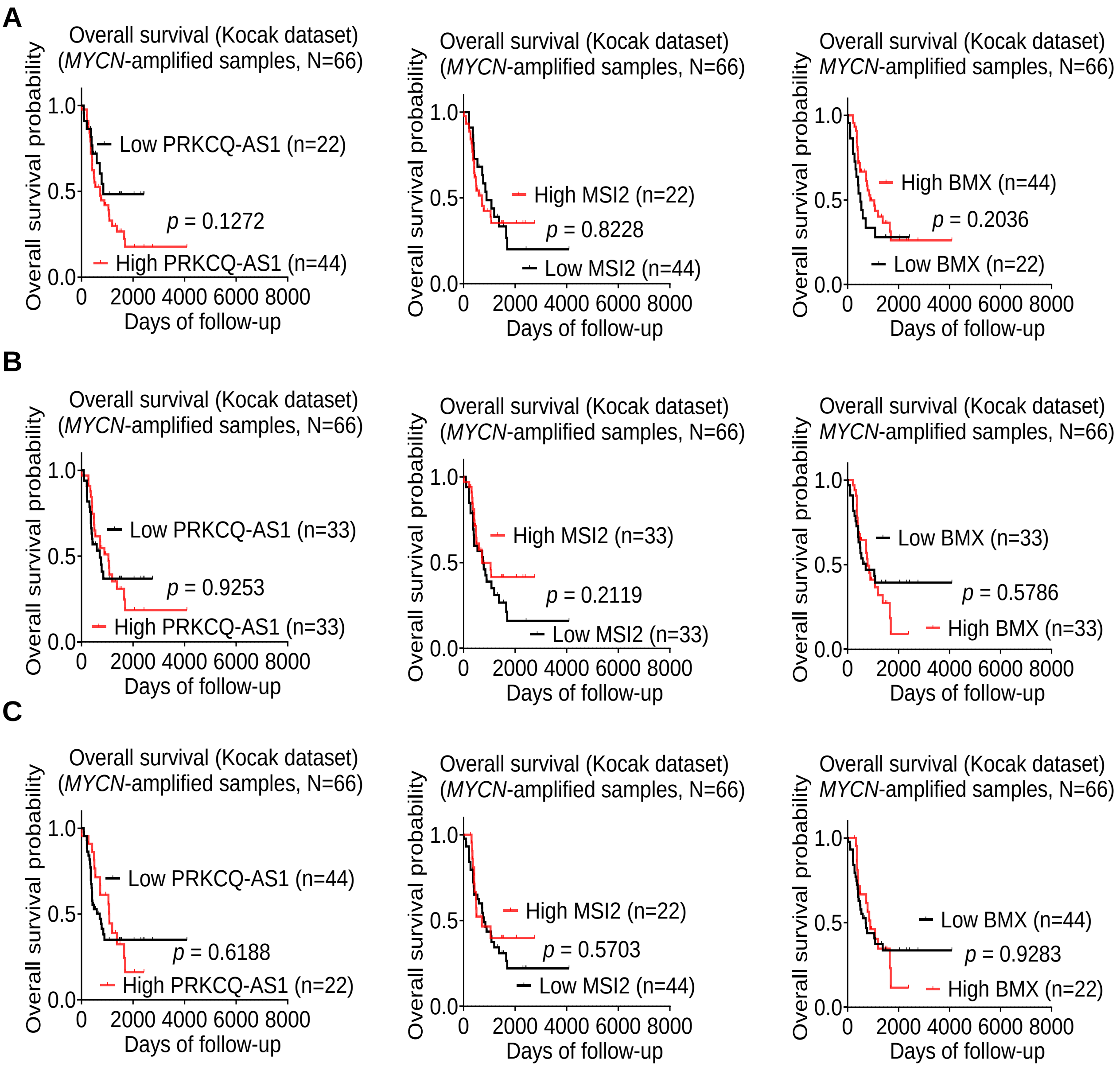

Figure S8. PRKCQ-AS1, MSI2 and BMX expression in *MYCN*-amplified human neuroblastoma tissues does not predict patient prognosis. (A-C) PRKCQ-AS1, MSI2 and BMX RNA expression in 66 *MYCN* gene-amplified human neuroblastoma tissues was extracted from the publicly available microarray gene expression Kocak dataset. Kaplan–Meier curves showed the probability of overall survival of patients according to the low quartile **(A),** the median **(B)** or the upper quartile **(C)** of PRKCQ-AS1, MSI2 or BMX expression. *P value* was obtained from two-sided log-rank tests.

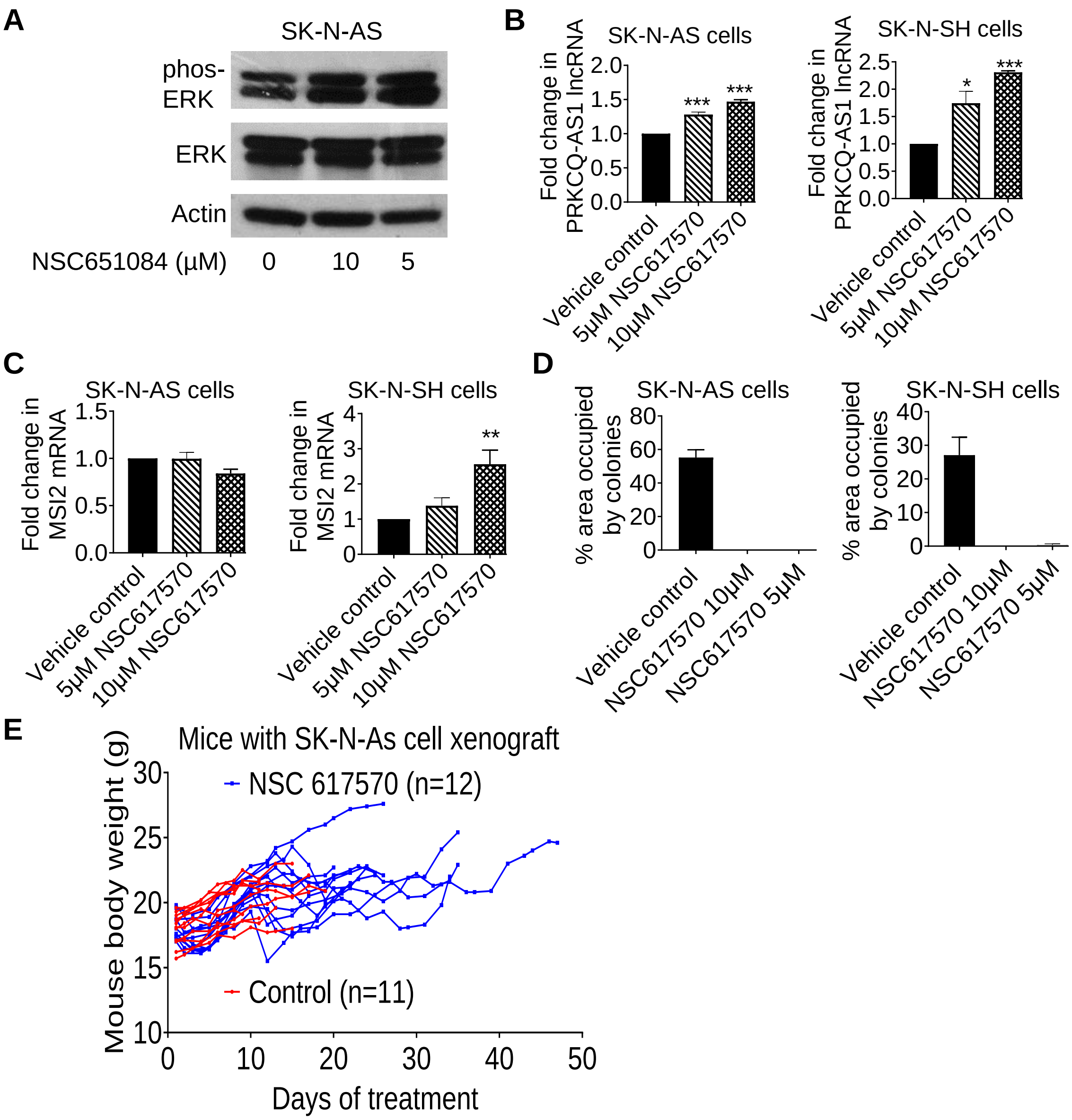

Figure S9. NSC651084 enhances ERK protein phosphorylation and NSC617570 significantly blocks neuroblastoma cell colony formation. (A) SK-N-AS cells were treated with vehicle control, 5μM or 10μM NSC 6511084 for 48 hours, followed by immunoblot analysis of total ERK protein (ERK) and phosphorylated ERK protein (phos-ERK). **(B-C)** SK-N-AS and SK-N-SH cells were treated with vehicle control, 5μM or 10μM NSC617570 for 48 hours, followed by RT-PCR analysis of PRKCQ-AS1 **(B)** or MSI2 **(C)** RNA expression. **(D)** SK-N-AS and SK-N-SH cells were treated with vehicle control, 5μM or 10μM NSC617570 for 14 (SK-N-AS) or 21 (SK-N-SH) days, followed by clonogenic assays. **(E)** SK-N-AS cells were xenografted into nude mice. When tumors reached 50 mm³, the mice were treated with NSC617570 at 8.75 mg/kg or vehicle control via i.p. injection, 5 times per week. Mice were culled when tumors reached 1000 mm^3, and mouse body weight was monitored. Each line represented one mouse. *In vitro* data were shown as the mean ± standard error of three independent experiments and evaluated by one-way ANOVA. *, ** and *** indicates $p < 0.05$, 0.01 and 0.001 respectively.
