# Supplementary material for: The Super Enhancer‐Driven Long Noncoding RNA PRKCQ‐AS1 Promotes Neuroblastoma Tumorigenesis by Interacting With MSI2 Protein and Is Targetable by Small Molecule Compounds
Source: Adv Sci (Weinh). 2025 Mar 18;12(18):2412520. doi: 10.1002/advs.202412520 (PMC12079515; doi:10.1002/advs.202412520)
Supplement: Supplementary file 8 — Supplemental Table 7 [file ADVS-12-2412520-s002.docx]

**Table S7. Sequences of primers used to generate PRKCQ-AS1 RNA fragments A – F and fragments 1 - 3**

| PRKCQ-AS1 Fragment A | F: TAATACGACTCACTATAGGGAGACCTCCTTACCAGCCACTTGT  R: ATTTAGGTGACACTATAGAAGGGGTTGAAAGCAGTCCCACCTT |
| --- | --- |
| PRKCQ-AS1  Fragment B | F: TAATACGACTCACTATAGGGAGACACTTCTGTCCTGGGGTCAT  R: ATTTAGGTGACACTATAGAAGGGAAGGAAGGATGCAAGACGTG |
| PRKCQ-AS1  Fragment C | F: TAATACGACTCACTATAGGGAGACCAGGACACGACTTTGCTTT  R: ATTTAGGTGACACTATAGAAGGGAGGGTAGGCCACTGTCCTCT |
| PRKCQ-AS1  Fragment D | F: TAATACGACTCACTATAGGGAGATAGAGGACAGTGGCCTACCC  R: ATTTAGGTGACACTATAGAAGGGGGGTAAGGTTTCCATTCACCT |
| PRKCQ-AS1  Fragment E | F: TAATACGACTCACTATAGGGAGATCGAAACCAGTGAAAGGTGA  R: ATTTAGGTGACACTATAGAAGGGGGTGTCAAGCAGTCCCCTAC |
| PRKCQ-AS1  Fragment F | F: TAATACGACTCACTATAGGGAGAGACTTAAAGGTTAAACAAATGATTTCC  R: ATTTAGGTGACACTATAGAAGGGAACCGTTTCTTGTTTAATTTCATTCT |
| PRKCQ-AS1  Fragment 1 | F: TAATACGACTCACTATAGGGAGATGGCCTCCTTACCAGC  R: ATTTAGGTGACACTATAGAAGGGTGCCCAAACAAATGCCT |
| PRKCQ-AS1  Fragment 2 | F: TAATACGACTCACTATAGGGAGACACTTCTGTCCTGGGGTCAT  R: ATTTAGGTGACACTATAGAAGGGAGTCCCACCTTGGCAATTGT |
| PRKCQ-AS1  Fragment 3 | F: TAATACGACTCACTATAGGGAGAGCTTTCAACTTTACTGTGCT  R: ATTTAGGTGACACTATAGAAGGGAAGGATGCAAGACGTGG |
