# Supplementary material for: The Super Enhancer‐Driven Long Noncoding RNA PRKCQ‐AS1 Promotes Neuroblastoma Tumorigenesis by Interacting With MSI2 Protein and Is Targetable by Small Molecule Compounds
Source: Adv Sci (Weinh). 2025 Mar 18;12(18):2412520. doi: 10.1002/advs.202412520 (PMC12079515; doi:10.1002/advs.202412520)
Supplement: Supplementary file 9 — Supplemental Table 8 [file ADVS-12-2412520-s008.docx]

**Table S8. Sequences of primers used to generate BMX mRNA CDS fragments**

| BMX CDS Fragment 1  (496 bp) | F: TAATACGACTCACTATAGGGAGAATGGATACAAAATCTAT  R: ATTTAGGTGACACTATAGAAGGGAAGGTGGGAACTC |
| --- | --- |
| BMX CDS Fragment 2  (499 bp) | F: TAATACGACTCACTATAGGGAGACCCAGACAGAGTGCT  R: ATTTAGGTGACACTATAGAAGGGACACTGTGTACATTC |
| BMX CDS Fragment 3  (488 bp) | F: TAATACGACTCACTATAGGGAGACCTTATTTAGTAA  R: ATTTAGGTGACACTATAGAAGGGAGCCATTGCTTAT |
| BMX CDS Fragment 4  (540 bp) | F: TAATACGACTCACTATAGGGAGAGCTTGCTGAAT  R: ATTTAGGTGACACTATAGAAGGGTCAATGCTTG |
